# For Whom Does Education Convey Health Benefits? A Two-Generation and Life Course Approach

**DOI:** 10.1177/00221465241249120

**Published:** 2024-06-04

**Authors:** Liying Luo, Lai Wei

**Affiliations:** 1 The Pennsylvania State University, University Park, PA, USA; 2 University of Hong Kong, Hong Kong, China

**Keywords:** controlled direct effect, education–health relationship, effect heterogeneity, two-generation setting

## Abstract

Scholars of social determinants of health have long been interested in how parent’s and own education influence health. However, the differing effects of parent’s and own education on health—that is, for what socioeconomic group education conveys health benefits—are relatively less studied. Using multilevel marginal structural models, we estimate the heterogeneous effects of parent’s and own education over the life course on two health measures. Our analysis considers both parent’s and respondent’s pre-education covariates, such as childhood health and socioeconomic conditions. We find that the protective effects of college completion against negative health outcomes are remarkably similar regardless of parent’s (measured by father’s or mother’s) education. Meanwhile, parent’s education has a larger effect when the average educational level is low in the population. Our results also reveal distinct life course patterns between health measures. We conclude by discussing the implications of our study for understanding the education–health relationship.

Voluminous research has documented the association between education and health (Cutler and Lleras-Muney 2008; Ross and Wu 1995; Warren et al. 2020). Studies suggested that both respondent’s own and their parent’s education influence health (Halpern-Manners et al. 2020; Hayward and Gorman 2004). However, prior research largely assumed that the effects of parent’s and own education on health are independent or additive. A few studies examined the potentially heterogeneous effects of parent’s and own education (see e.g., Andersson 2016; Bauldry 2014; Ross and Mirowsky 2011), but the empirical evidence is mixed. Moreover, they often focused on respondents’ characteristics but not their parent’s childhood health or early life socioeconomic conditions. Addressing these limitations will improve the estimation of education effects on health in a two-generation setting.

Our study makes three contributions. First, we examine a key yet understudied question about the education–health relationship: Whom does education benefit? Although greater education is believed to convey health benefits, such health benefits may vary in degree by socioeconomic backgrounds. The resource substitution hypothesis, for example, suggests that individuals who are from a disadvantaged background may benefit more from college education (Ross and Mirowsky 2011). By contrast, the resource multiplication perspective (Ross and Mirowsky 2006) indicates that the health return to education is largest for those whose parents also have higher levels of education. We test these hypotheses utilizing the multigenerational data from the Panel Study of Income Dynamics (PSID).

In assessing effect heterogeneity, we pay attention to educational and health selections that could subject analytical results to alternative interpretations. Specifically, does education primarily affect health (the social causation hypothesis; Warren 2009), or does health affect educational attainment (Haas 2006)? We utilize multigenerational data from the PSID to account for intergenerational selection to better understand the nature of the education–health relationship across generations. The PSID is uniquely suitable for our research goals because it provides key pre-education information, such as childhood health and socioeconomic conditions, for both the parent and the respondent generations. Such multigenerational data are critical for refining the estimation of education effects, especially parent’s education effect, on health.

We also pay attention to the life course differences in parent’s and own education effects. Although health changes with age, education effects on health may also differ by age (Carr 2019; House et al. 1994). For example, the “cumulative disadvantage theory” (Dannefer 2020; O’Rand 1996) predicts that education effects are greater in middle and older ages than younger ages. Examining life course differences in the education–health relationship may also provide clues for reconciling the mixed findings in prior research. Our results suggest that the mixed results are partly attributable to different age samples across studies.

Second, we conduct a novel analysis to examine at the population level if the influence of parent’s education on health is a function of the average education levels. The percentage of Americans age 25 or older completing high school increased from 55.2% in 1970 to 90.9% in 2020, and the percentage completing bachelor’s degrees increased from 11.0% to 37.5% for the same period of time (National Center for Education Statistics 2022). What are the implications of the rising education levels for the influence of socioeconomic background on health? Would the influence of parental education be greater or smaller as the average level of education increases among younger generations? Distinct from the individual-level effect heterogeneity discussed earlier, understanding this population-level heterogeneity may advance knowledge about the education–health relationship by conjugating individual educational attainment and population educational compositions.

Third, we describe a methodological framework for studying the education–health relationship in a two-generation setting. Although researchers are increasingly aware of the limitations of the standard regression model for addressing selection issues, alternative methods and estimands that are more suitable in a multigeneration setting are less discussed (Lundberg, Johnson, and Stewart 2021). We explicate the interdependent causal pathways of education effects on health and propose well-defined causal estimands for estimating education effects on health. Compared to the standard regression, this approach improves the assessment of education effects by accounting for pre-education factors, including educational and health selections across generations. Also, one proposed estimand—the controlled direct effect of parent’s education—is particularly instrumental for informing the second research question about the relative importance of family backgrounds as education levels increase in younger generations.

Here, we focus on one type of effect heterogeneity in the education–health link (i.e., heterogeneous education effects by parental backgrounds). We are cognizant of other important heterogeneity, including differing education effects among health measures (for a review, see e.g., Cutler and Lleras-Muney 2008) and by race, ethnicity, and gender (Andersson 2016; Daw, Margolis, and Verdery 2015; Umberson et al. 2014). We provide discussions about other types of heterogeneity and encourage future studies to examine other health outcomes and multiple sociodemographic heterogeneity in the education–health relationship.

The remainder of this article is organized as follows. Following a brief review of the education–health literature, we discuss reasons for expecting parental and own education to interact with each other to affect health over the life course. We next explicate why coefficients from the conventional regression model do not represent the kind of education effects that researchers intend to estimate. We then describe our estimands and models. We analyze the multigenerational data from the PSID to estimate the effects of parental and own education on self-reported health status and severe psychological distress. We report the findings and conclude by discussing the policy implications, limitations, and future directions.

## Background

### Parent’s and Own Education Effects on Health

A great deal of research has documented the educational gradients in health outcomes and behaviors (Cutler and Lleras-Muney 2008; Hummer and Hernandez 2013; Mirowsky and Ross 2003). That is, greater education is associated with better health, lower mortality, and fewer risky health behaviors. This education–health relationship is largely robust and persistent in time—, although its strength varies across health measures (Cutler and Lleras-Muney 2008; Phelan, Link, and Tehranifar 2010).

The direction of the education–health association has gathered scholarly attention. For example, the health selection hypothesis suggests that because early life health influences individuals’ ability to attain education and other socioeconomic resources, it is health that primarily affects education (Blane, Smith, and Bartley 1993; Haas 2006). By contrast, the social causation hypothesis indicates an opposite direction, that is, education primarily affects health (Grundy and Sloggett 2003; Warren et al. 2020). Link and Phelan (1995) developed the “fundamental cause theory” to conceptualize mechanisms and pathways that socioeconomic status (SES) such as education may lead to better health. They posit that SES is linked to health and mortality through various mechanisms, including health knowledge, behaviors, and resources.

Meanwhile, although much education–health research focuses on respondents’ own education, the social network literature suggests that parent’s education may also influence health through similar mechanisms, including knowledge, exposure, and resources that are shared and/or transmissible within family and across generations (Daw et al. 2015; Halpern-Manners, Hernandez, and Wilbur 2022; Pescosolido 2006). For example, better educated parents may be more aware of the negative consequences of and less likely to engage in risky health behaviors, such as smoking and substance use, and thus reduce their children’s exposure to these behaviors. They also have more economic and social resources to help their children develop and sustain healthier lifestyles. Health behaviors and conditions that form in childhood may persist as the person ages, leading to distinct age-graded trajectories in health.

Using multigenerational data, we seek to test the following hypotheses:

*Hypothesis 1a:* After accounting for selection, both parental and own education influence health.
*Hypothesis 1b:* After accounting for selection, parental and own education do not affect health.

### For Whom Does Education Convey Health Benefits?

Researchers also debate about whether education conveys uniform or differential benefits by parental backgrounds. For example, the resource multiplication perspective (Ross and Mirowsky 2006) indicates that health returns to education are larger for those whose parents also have higher levels of education. This is because the resources that the advantaged group has may multiply to augment their socioeconomic advantages. For example, individuals whose parents were college educated may obtain more knowledge about higher education from their parents and parents’ network, which helps with college application, adjusts expectations, and supports achievement of educational goals. They are subsequently better equipped to navigate the system and deal with stress while in college. Upon and after graduation, individuals with greater parental education have more social capital and economic opportunities to realize greater returns to their investment in education, which, in turn, could be used to support healthy lifestyles.

Alternatively, the resource substitution hypothesis posits that because multiple socioeconomic resources are related to health, well-resourced people can substitute one that is lacking with another to support health and reduce disease risk (Mirowsky and Ross 2003; Ross and Mirowsky 2011). It implies that for those whose parents have more education and thus more socioeconomic resources, less own education (i.e., lacking one specific resource) may not be detrimental because they could substitute it with higher levels of family income and networks. When parents have less education and fewer education-related socioeconomic resources, their children may be more dependent on their own education for their health. As a result, the effect of greater education, or lack thereof, can be more salient than it is for those with greater parental education.

At the same time, because social mobility disrupts social ties and networks, the health benefits of greater attained education for those from a disadvantaged background may be reduced. For example, Sorokin’s (1927) disassociative theory posits that moving from one SES to another interrupts social networks and incurs stress due to difficult acculturation and social rejection. It implies that greater own education may not be as beneficial for individuals from a disadvantaged background as for the advantaged (House, Landis, and Umberson 1988; Luo 2022). Relatedly, individuals with less parental education may have fewer network resources to obtain well-paying jobs, which, in turn, could lead to negative health.

Intriguingly, there are two mobility-related mechanisms that could reduce or offset the network disruption’s negative impacts for the socioeconomically disadvantaged. First, greater education, particularly a college degree, is considered an important milestone across socioeconomic spectrums. However, it may be viewed as an even more special and significant accomplishment for those from a disadvantaged background because college aspiration and completion are not parents’ expectation or the norm in their social circles. As a result, the upwardly mobile individuals may experience a high level of sense of achievement because they have overcome challenges and obstacles from disadvantaged early life circumstances. Because the sense of fulfillment and achievement is an important mechanism by which more education could lead to better general health and well-being (Murray-Harvey 2010), the upward mobility experienced by those from a disadvantaged background may subsequently have a particularly strong positive effect on their health.

Second, less educated and less resourced groups are often burdened with higher prevalence of risky behaviors and negative health outcomes, such as smoking and obesity. Because health behaviors spread through social networks (Christakis and Fowler 2007, 2008; Daw et al. 2015), upwardly mobile people may be less exposed to health risk by extricating themselves from the “old” networks in which there are higher rates of smoking, sedentarism, substance use, alcohol consumption, and other unhealthy behaviors. Meanwhile, higher education may also imply exposure to new network ties that promote and normalize healthier lifestyles. In other words, although network disruption decreases available social support, it may operate to insulate the upwardly mobile from health risk while exposing them to a healthier environment.

Importantly, such disruption of “old” ties and exposure to “new” environment begins in young adulthood. From the life course perspective, during these critical and formative ages, individuals are less resistant to and more motivated to abide by and uphold new behaviors and lifestyles, leaving lasting positive influence on health into midlife and older ages.

Empirical evidence regarding the heterogeneous health returns to education is mixed. For example, Ross and Mirowsky (2011), Schaan (2014), and Vable et al. (2018) found that higher education provided more health benefits for those from a disadvantaged background. By contrast, Bauldry (2014), Settels (2022), and Veenstra and Vanzella-Yang (2022) suggested that college education is more beneficial for the advantaged. Such mixed findings may be attributed to various sources, including different methodological approaches, age samples, and/or health outcomes. Reconciling the mixed results requires meta-analysis that is beyond the scope of the current study. However, we discuss how our life course analysis may provide clues for understanding such mixed findings.

In short, there may be distinct mechanisms generating heterogeneous education effects on health by socioeconomic backgrounds. We seek to test the following competing hypotheses about for whom greater education conveys health benefits:

*Hypothesis 2a:* Greater own education has larger health benefits for individuals from an advantaged background.
*Hypothesis 2b:* Greater own education has larger health benefits for individuals from a disadvantaged background.

### Life Course Differences

Although health changes with age, research shows that the magnitude of the education effect on health may also differ across ages. For example, the “cumulative advantage/disadvantage” theory posits that the educational inequality in health may be larger in later life (Dannefer 2020; O’Rand 1996). College-educated individuals whose parents are also highly educated can avail themselves of more family resources and networks to maintain good health. Meanwhile, the initial disadvantages of individuals from families with less education and fewer socioeconomic resources may cumulate as one ages. As a result, educational inequality in health may widen over the life course.

An important yet less noted life course mechanism for expecting larger educational gradients in health in middle and older ages is adult children’s caregiving responsibilities for elderly parents, a life stage termed the “sandwich” or “stretched” generation (Rogerson and Kim 2005). Low SES parents are at higher risks of various health conditions and diseases, which increases the likelihood of their dependence on adult children for caregiving and economic resources. It implies that the educational gradients may be larger in middle or older ages than in younger ages, especially for those from disadvantaged backgrounds. We thus hypothesize that:

*Hypothesis 3:* Education effects on health are more pronounced in older ages than in younger ages.

### Parent’s Education Effect as a Function of the Average Educational Levels

Compared to effect heterogeneity by family backgrounds, even less research examines how parental education effects may differ by average education levels in the population. The percentage of Americans age 25 or older completing high school rose from 55.2% in 1970 to 90.9% in 2020, and the percentage completing bachelor’s degrees increased from 11.0% to 37.5% for the same period of time (National Center for Education Statistics 2022). Such rising education levels among the younger generations require an updated understanding about the role of parent’s education in influencing their adult children’s health. Specifically, compared to a scenario of low education, should we expect greater or smaller influence of parent’s education on their children’s health when the average education level is higher? Understanding this question has policy implications for educational health inequalities. If, for example, low parental education is less consequential when the average educational level in a population is higher, then programs and policies that provide more educational opportunities and resources could be effective for addressing health disparities by socioeconomic backgrounds.

Although the resource substitution and multiplication hypotheses are mostly tested at the individual level, they are also suggestive for assessing population-level effect heterogeneity. According to the resource substitution hypothesis, because greater own education may compensate early life disadvantages, parent’s less education likely has a smaller impact on health as the average education level increases across generations. By contrast, the resource multiplication hypothesis predicts that because multiple resources reinforce each other, the influence of parent’s education is expected to be more pronounced with rising education levels. Using multigenerational longitudinal data, we test the following hypotheses:

*Hypothesis 4a:* The influence of parent’s education on health and well-being will be smaller when the average education level among the respondents is higher.
*Hypothesis 4b:* The influence of parent’s education on health and well-being will be greater when the average education level among the respondents is higher.

The strength of the education–health association and its mechanisms seem to vary by health measures (for a review, see e.g., Cutler and Lleras-Muney 2008). We focus on two health measures, namely, self-reported health and psychological distress, for the following considerations. First, self-reported health and psychological well-being are two of the most studied health outcomes in the social determinants of health literature. Second, despite its unique multigenerational design, the empirical data that we analyze here were not originally designed as a health study. Self-reported health and psychological distress are among the limited health measures that have been consistently collected across waves and across generations.

## Data and Methods

### Data and Measures

We analyzed the 1968 to 2017 data from the Panel Study of Income Dynamics (PSID), a nationally representative survey of more than 5,000 families in the United States since 1968. The PSID was suitable for the present study for two reasons. First, the PSID is a longitudinal study that permits estimating education effects on health over the life course. Second, the PSID collects data from multiple generations, so a rich set of pre-education variables (i.e., variables operating before completing one’s education) for both the parent’s and the respondent’s generations are available. For example, the PSID provides information not only about the respondents’ early life circumstances but also their parents’ parental education, childhood health, and socioeconomic conditions. Controlling for such pretreatment variables is critical for estimating the causal effects of parental and own education. In addition, the control variables are based on the same questions across generations, allowing symmetric control variables for both generations. Symmetric controls are instrumental in that the difference between estimated parent’s and own education effect should not be attributed to different control variables. By contrast, other studies, including the National Longitudinal Study of Adolescent to Adult Health and the National Longitudinal Study of Youth cohorts, often lack pre-education variables for the parent’s generation, including the parent’s early childhood health, socioeconomic conditions, and educational levels. In short, although not originally designed as a health study, the PSID’s unique multigenerational design offers opportunities to improve estimation of the effects of parent’s and own education on health.

As mentioned earlier, only a limited set of health outcomes has been consistently measured over time and across generations in the PSID. We considered two health outcomes, namely, self-report health and psychological distress, both of which are measured longitudinally across generations. Self-reported health was a binary variable with respondents reporting poor or fair health coded 1 and 0 otherwise. Severe psychological distress was also a binary variable based on the Kessler 6 score, with 13 or higher scores coded 1 and 0 otherwise. Alternative measures of general health (e.g., a continuous variable ranging from 1 to 5) and psychological distress (e.g., moderate distress defined as a Kessler 6 score ≥5 [Prochaska et al. 2012] or a Kessler 6 score as a continuous variable) were also used, and the results (see Tables 5S and 6S in the online version of the article) were largely consistent.

The key predictors were the respondent’s own and their parent’s education levels. Based on the respondents’ and their parents’ self-reported years of schooling, we constructed three education categories for each generation: less than high school (<12 years of schooling), high school completion (12–15 years), and college completion (≥16 years) or above. We acknowledge possible discrepancy between education completion/degree and these cutoff points for years of schooling. The main analysis used father’s education; when their own report was missing, their child’s report was used. We considered parental education based on mother’s education in additional analyses. The results were largely similar using the two measures of parental education (see Supplement Figures 1S to 3S in the online version of the article). We provide discussions about the two parental education measures in the discussion section.

We restricted the sample to adult participants who were age 18 to 70. We next followed the PSID’s instruction for multigenerational linkage (see R code in the Technical Appendix in the online version of the article) to obtain 10,259 unique parent–child pairs. Among these parent–child pairs, we further restricted the sample to those with complete information about own and their father’s and mother’s education and also at least one measure of general health or psychological distress. About 60% to 70% of the parent–child pairs successfully linked were included in the final samples. The final sample for self-reported health and psychological stress included 6,405 and 7,156, respectively, parent–child pairs, with an average of six observations per respondent (i.e., child in a parent–child pair). Table 1 reports the descriptive statistics of key variables.

**Table 1.** Descriptive Statistics for Key Analytic Variables, the Panel Study of Income Dynamics, 1968–2017.

	Self-Reported Health				Psychological Distress			
Description	Mean	SD	Minimum	Maximum	Mean	SD	Minimum	Maximum
Health outcome	.10	.30	0	1.00	.03	.18	0	1.00
Father’s educational attainment (3 = college completion, 2 = high school, 1 = less than high school)	1.91	.71	1.00	3.00	2.04	.67	1.00	3.00
Respondent’s educational attainment (3 = college completion, 2 = high school, 1 = less than high school)	2.24	.58	1.00	3.00	2.30	.58	1.00	3.00
Age at the time of survey	35.14	10.76	18.00	70.00	35.13	12.79	18.00	70.00

*Note:* Descriptive statistics are unweighted. Analyses of self-reported health status and psychological distress are based on different samples. For general health status, the number of unique father–child pairs is 6,405, and the total number of observations is 37,398. For psychological distress, the number of unique father–child pairs is 7,156, and the total number of observations is 39,725.

Table 2 provides descriptive statistics of father- and respondent-level covariates. For both generations, we considered demographic characteristics, such as the person’s race, gender, and regions growing up. We also considered childhood socioeconomic conditions and childhood health, such as childhood mental health, substance use, childhood psychological well-being, and coresidence status with parents. Because the PSID contains respondents from multiple birth cohorts, we also included 10-year birth cohort indicators as a covariate in all analyses. We included additional analysis for each cohort (see Appendix Tables 15S to 18S in the online version of the article), and the results were largely consistent across birth cohorts.

**Table 2.** Descriptive Statistics for Pre-education Control Variables for Respondents and Fathers, the Panel Study of Income Dynamics, 1968–2017.

	Self-Reported Health					Psychological Distress				
	Distribution					Distribution				
Respondent’s Characteristics	1	2	3	4	5	1	2	3	4	5
Gender (1 = male, 2 = female)	.50	.50				.50	.50			
Race (1 = white, 2 = other)	.67	.33				.67	.33			
Ever had depression before 17 years old (1 = yes, 2 = no)	.06	.94				.06	.94			
Drug or alcohol problems before 17 years old (1 = yes, 2 = no)	.03	.97				.03	.97			
General health status before 17 years old (1 = excellent, 2 = very good, 3 = good, 4 = fair, 5 = poor)	.53	.29	.13	.04	.01	.54	.29	.13	.04	.01
Other psychological problem before 17 years old (1 = yes, 2 = no)	.02	.98				.02	.98			
Region growing up (1 = Northeast, 2 = North, 3 = South, 4 = West)	.15	.28	.40	.17		.16	.28	.40	.16	
Type of residence growing up (1 = farm, 2 = suburb, 3 = large city, 4 = other)	.09	.56	.33	.03		.09	.56	.32	.03	
Parental economic well-being growing up (1 = well off, 2 = average, 3 = poor)	.21	.47	.32			.20	.47	.32		
Living with parents growing up (1 = no, 2 = yes)	.19	.81				.19	.81			
	Mean	SD	Minimum	Maximum		Mean	SD	Minimum	Maximum	
Age	35.14	10.76	18	70		35.13	12.79	18	70	
	Distribution					Distribution				
Father’s Characteristics	1	2	3	4	5	1	2	3	4	5
Race (1 = white, 2 = other)	.67	.33				.67	.32			
Ever had depression before 17 years old (1 = yes, 2 = no)	.01	.99				.01	.99			
Drug or alcohol problems before 17 years old (1 = yes, 2 = no)	.01	.99				.02	.98			
General health status before 17 years old (1 = excellent, 2 = very good, 3 = good, 4 = fair, 5 = poor)	.61	.23	.13	.02	.01	.61	.23	.13	.02	.01
Other psychological problem before 17 years old (1 = yes, 2 = no)	.01	.99				.01	.99			
Region growing up (1 = Northeast, 2 = North, 3 = South, 4 = West)	.16	.29	.38	.17		.16	.29	.37	.18	
Type of residence growing up (1 = farm, 2 = suburb, 3 = large city, 4 = other)	.19	.45	.34	.02		.19	.45	.33	.02	
Parental economic well-being growing up (1 = well off, 2 = average, 3 = poor)	.39	.44	.17			.40	.44	.17		
Living with parents growing up (1 = no, 2 = yes)	.23	.77				.23	.77			

*Note:* Descriptive statistics are unweighted after imputation. For general health status, the number of unique father–child pairs is 6,405, and the total number of observations is 37,398. For psychological distress, the number of unique father–child pairs is 7,156, and the total number of observations is 39,725.

Missing values in control variables were imputed using the R package *mice*. Specifically, a missing covariate was imputed using a model with all other control variables as predictors. Continuous, binary, and categorical variables were imputed using, respectively, predictive mean matching, logistic regression imputation, and polytomous regression imputation. We conducted a robustness check by comparing results with and without imputing control variables (see Tables 3S and 4S in the online version of the article). The main findings appeared similar with and without imputation despite numeric differences.

### Conceptual Framework in a Two-Generation Setting

We began by clarifying the interdependent pathways through which parent’s and own education may influence the respondent’s health. This clarification helps readers understand why the standard regression approach is not suitable for quantifying education effects on health in two-generation settings. It also serves as a conceptual model for motivating and defining our estimands. We then described the identification and estimation procedures of the estimands using weighted multilevel marginal structural models.

**Figure 1.**
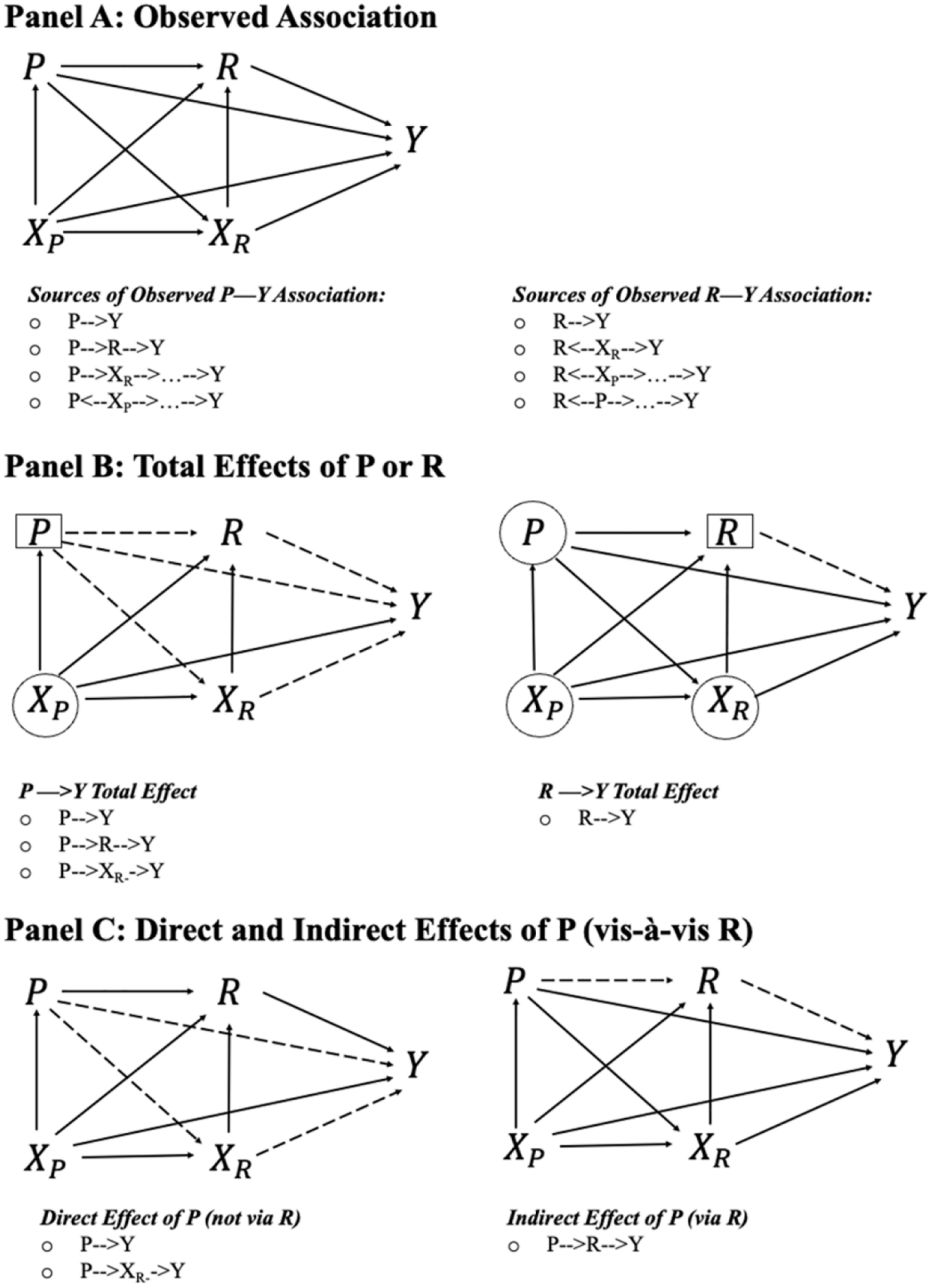
Causal Quantities of Interest in Assessing Parent’s and Own Education Effect on Health. Panel A: Observed Association. Panel B: Total Effects of P or R. Panel C: Direct and Indirect Effects of P (vis-à-vis R). *Note:* Figures are directed-acyclic graphs. An arrowhead line represents a causal link. P, R, X_P_, X_R_, and Y denote, respectively, parent’s education, respondent’s own education, parent-level pre-education covariates, respondent-level pre-education covariates, and the outcome of interest. Variables in rectangles in Panel B are predictors in a statistical model, and variables in circles are control variables. Dashed lines are causal paths of interest.

The directed-acyclic graphs (DAGs) in Figure 1 illustrate the conceptual paths and causal quantities that may interest education-health researchers in a two-generation setting. An arrowhead line represents a causal link. Let *P* be parent’s education; *R* denotes respondent’s own education; *X_P_* and *X_R_* are parent- and respondent-level covariates including demographics, childhood socioeconomic, and health conditions; and *Y* is the outcome, such as health status and psychological distress. Figure 1, Panel A shows that the observed relationship between parental education and health may be attributed to multiple (causal and noncausal/confounding) sources, including four causal pathways *P* → *Y*, *P* → *R* → *Y*, *P* → *X_R_* → *Y*, and *P* → *X_R_* → *R* → *Y*, and multiple confoundings through *X_P_* (*P* ← *X_P_* → . . . → *Y*). The observed relationship between own education and health also includes the causal pathway of *P* → *Y*, and various noncausal sources through *X_R_*, such as *R* ← *X_R_* → *Y*, *R* ← *X_P_* → . . . → *Y*, and *R* ← *P* → . . . → *Y*.

The DAGs in Figure 1, Panel A clarify that first, estimating the effect of own education on health (*R* → *Y*) requires accounting for two selections. One is the intergenerational selection on education represented by the *P* → *R* path. The social stratification and mobility literature shows that children born to well-educated parents are more likely to obtain a college degree than those whose parents have less education (Halpern-Manners et al. 2020). The other selection involves the *X_R_* → *R* pathway from childhood health and socioeconomic conditions to educational attainment. Childhood biopsychosocial factors influence educational opportunities and attainment (Haas 2006). It is thus critical for education–health researchers to control the education selection (*R* ← *P* →*Y*) and health–education selection (*R* ← *X_R_* →*Y*).

Figure 1, Panel A also provides intuitions for a methodological dilemma when simultaneously examining parental and own education effects on health in the standard regression model. On the one hand, estimating own education effect *R* → *Y* requires controlling for *P*, *X_P_*, and *X_R_*. On the other hand, estimating parental education effect *P* → *Y* requires not controlling for *R* or *X_R_*. This is because the standard regression model with *R* or *X_R_* as predictors will incorrectly exclude the two indirect causal pathways of parental education on health *P* → *X_R_* → *Y* and *P* → *R* → *Y*. It also erroneously induces two noncausal pathways *P* ← *X_P_* → *X_R_* → *Y* and *P* ← *X_P_* → *R* → *Y* where *X_R_* operates as a collider. Conceptually, the two indirect causal pathways imply that parent’s education *P* would lead to different childhood health and socioeconomic conditions for the respondent *X_R_*, differing own education *R*, and subsequently their health in adulthood *Y*. That is, we should include the indirect pathways *P* → *X_R_* → *Y* and *P* → *R* → *Y* in parental education effects. However, controlling for *X_R_* and *R* in the standard regression model, as often done in prior research, excludes or “controls away” such indirect effects from the *P* → *Y* total effect. The marginal structural models that we discuss is instrumental for addressing this methodological dilemma.

Before defining our estimands, we clarify that we focus on the overall effects of education on health because education is a critical life course milestone that often (although not always) temporally proceeds other events, such as occupation and marriage, and structures subsequent socioeconomic opportunities. Methodologically, we did not consider posttreatment variables, such as income, occupation, and family, that occur after education completion because including such posteducation variables will bias the estimation of education effect (Pearl 2022).

### Four Causal Estimands

Drawing on the literature on causal mediation (Robins and Greenland 1992), we proposed to assess the effect of parental and own education with four well-defined causal estimands. We describe the definition and identification of these estimands below; see the Technical Appendix in the online version of the article for additional discussions including a comparison with other causal estimands.

We used the average treatment effects (ATEs) to assess the total effect of parental and own education, respectively, on health: *ATE_p_ = E[Y(p) – Y(p′)]* (Estimand 1) and *ATE_R_ = E[Y(r) – Y(r′)]* (Estimand 2). Panel B in Figure 1 clarifies that the identification of parental education effect can be achieved by conditioning on *X_P_*, and own education effect can be identified by conditioning on *P*, *X_P_*, and *X_R_*. Because we are also interested in how these effects may change over the life course, we estimated the age-specific analogs of these estimands: *E[Y(p) – Y(p′)|age]* and *E[Y(r) – Y(r′)|age]*.

To examine for what parental education group greater own education conveys health benefits, we estimated the conditional ATE (CATE) of own education by parental education groups, *CATE_R|P_ = E[Y(r) – Y(r′)|p]* (Estimand 3). The interpretation of the CATE is comparable to that of an interaction effect in the conventional regression model. Like the ATE, the CATEs of own education are identifiable assuming that all *X_P_* and *X_R_* are observed. To assess the life course differences of own education effects, we estimated the age-specific analogs of the CATEs: *E[Y(r) – Y(r′)|p,age]*.

To investigate if the effects of parental education is a function of the education levels of the younger generation, we defined and estimated the controlled direct effect (CDE) of parental education on health, *CDE_p|r_ = E[Y(p, r) – Y(p′, r)]* (Estimand 4). The meaning and interpretation of CDEs differ from an interaction effect in the standard regression model. Specifically, the CDEs quantify the effect of a predictor (e.g., parental education) with the value of the mediator (e.g., own education) fixed at a value uniformly in the population. In a two-generation setting, the CDEs express the effect of parental education on health in hypothetical scenarios in which all respondents complete college, high school, or less than high school education. The CDEs are informative by providing initial but critical evidence about the relative importance of socioeconomic backgrounds as a function of educational compositions in the population. To assess the life course changes in parental education effects, we estimated the age-specific analogs of the CDEs: *E[Y(p, r) – Y(p′,r)|age]*.

We clarify that the CDEs of parental education are distinct in two ways from the CATEs of the respondent’s own education on health by parental educational levels. Substantively, the CDEs of parental education provide a novel assessment of the influence of parental education in different counterfactual scenarios of educational compositions in the population. By comparison, the CATEs of own education assess at the individual level, greater education conveys similar or differing health benefits by parent’s education levels. Methodologically, the estimation of the CDEs and CATEs requires distinct sets of pretreatment control variables to avoid posttreatment or collider bias and should be estimated in separate statistical procedures.

### Weighted Multilevel Marginal Structural Models

We estimated the estimands in two steps. Step 1 is to estimate a series of covariate balancing propensity scores (CBPS). CBPS is a type of propensity score that optimizes the covariate balance between treatment groups. It has been shown to outperform traditional propensity scores in estimating causal effects (Imai and Ratkovic 2014). The weights for the parental education effect model and the own education effect model were ${w^{P}}_{i}=\frac{I\left(P_{i}=p\right)}{\hat{p}(P_{i}|X_{P,i})}$ and ${w^{R}}_{i}=\frac{I(P_{i}=p,R_{i}=r)}{\hat{p}(P_{i}|X_{P,i})\hat{p}(R_{i}|X_{P,i},X_{R,i})}$ , where $\hat{p}(P_{i}|X_{P,i})$ and $\hat{p}(R_{i}|X_{P,i},X_{R,i})$ are the CBPS estimates for parental education and own education. We computed the CBPS using the R package *CBPS*.

We used propensity score weighting instead of propensity score matching in our analysis for two reasons. First, weighting uses data more efficiently in the sense that unlike matching, it does not exclude observations. Second, matching may be unwieldy when the treatment variables are multinomial, as they are in the current study. Evidence from simulations and empirical studies largely suggests few differences in results between the two methods (Austin and Stuart 2017; Elze et al. 2017). However, recent research points out some concerning properties of the propensity score as a balancing metric and suggests using propensity score weighting instead (King and Nielsen 2019).

In Step 2, we used the CBPS weights to estimate a series of multilevel marginal structural models (MMSMs). This weighted MMSM approach accounts for within-person dependence in longitudinal data. Specifically, we fit the following four weighted MMSMs using the R package *lme4:*

(1) $$Y_{ia}=\beta_{0}+\beta_{1}P_{i}+\beta_{4}cohort_{i}+\mu_{i}+e_{ia},w=w^{P};$$

(2) $$Y_{ia}=\beta_{0}+\beta_{1}R_{i}+\beta_{4}cohort_{i}+\mu_{i}+e_{ia},w=w^{R};$$

(3) $$\begin{array}{l} Y_{ia}=\beta_{0}+\beta_{1}P_{i}+\beta_{2}R+\beta_{3}P_{i}*R_{i}\\ +\beta_{4}cohort_{i}+\mu_{i}+e_{ia},w=w^{P}\times w^{R}; \end{array}$$

(4) $$\begin{array}{l} Y_{ia}=\beta_{0}+\beta_{1}P_{i}+\beta_{2}R_{i}+\beta_{3}P_{i}*R_{i}\\ +\beta_{4}cohort_{i}+\mu_{i}+e_{ia},w=w^{R}; \end{array}$$

where *Y_ia_* is the health outcome for person *i* at age *α*; *β_1_*, *β_2_*, and *β_3_* are regression coefficients for parent’s education, own education, and the parent’s–own education interaction terms, respectively; and *µ* and *e* are individual-specific random effect and response-specific idiosyncrasies. A birth cohort indicator was included as a control because educational attainment and the influence of education on health may differ across cohorts (Lynch 2003). Because observations were nested within participants, we included individual-level random intercepts to account for dependency among multiple observations within participants. The *β_1_*’s in Equations 1 and 2 give total effect estimates (i.e., ATEs of parental and own education effects). Linear combinations of *β_1_*’s and *β_3_*’s in Equation 3 and linear combinations of *β_2_*’s and *β_3_*’s and Equation 4, respectively, provide CDE estimates of parental education and CATE estimates of own education.

To assess how the two education effects may change over the life course, we estimated another set of MMSMs with respondent random intercepts as follows:

(5) $$Y_{ia}=\beta_{0i}+\beta_{1i}a_{i}+\beta_{2i}{a_{i}}^{2}+e_{ia},$$

where *Y_ia_*, *β_0i_*, *β_1i_*, *β_2i_*, and *e_ia_* are defined as in Equations 1 through 4. All three coefficients are allowed to vary for individuals with different own or parental education levels:

(6) $$\begin{array}{l} \beta_{0i}=\gamma_{00}+\gamma_{01}Z_{HighSchool}+\gamma_{02}Z_{College}\\ +\gamma_{03}Cohort_{i}+\mu_{0i}, \end{array}$$

(7) $$\beta_{1i}=\gamma_{10}+\gamma_{11}Z_{HighSchool}+\gamma_{12}Z_{College},$$

(8) $$\beta_{2i}=\gamma_{20}+\gamma_{21}Z_{HighSchool}+\gamma_{22}Z_{College},$$

where *Z* is a dummy variable indicating parental or own education levels, s are education fixed effects, and is individual-specific random effect. To estimate parental education effects, we weighted these equations by *w^P^_i_*. To estimate own education effects, we weighted the equations by *w^R^_i_*.

The meaning of and socioeconomic and health returns to education may differ for different birth cohorts (Lynch 2003). In the main analysis, we included cohort fixed effects to account for the marginal differences between cohorts. In an additional analysis, we examined if the main conclusions differ by cohorts by including complex interaction terms, such as cohort-specific education effects and cohort-specific life course trajectories. These results were largely consistent with our main findings about the relationship between health and parental and own education for each cohort (see Supplement Tables 15S–18S and Figure 5S in the online version of the article), although some estimates became statistically insignificant due to smaller samples sizes in cohort-specific subsample analyses.

In sum, to estimate the average and potentially differing effects of parent’s and own education on health, we specified four causal estimands that were motivated by a conceptual framework in a two-generation setting. We used a series of MMSMs weighted by CBPS that account for pre-education covariates for both generations and within-respondent correlation. Compared to other causal inference methods for observational data, such as propensity score matching, this CBPS-MMSM approach is advantageous in that it (a) does not exclude observations and thus uses the data more efficiently, (b) is more suitable for binary outcomes analyzed in the current research, and (c) accounts for correlation among observations within respondents.

## Results

**Figure 2.**
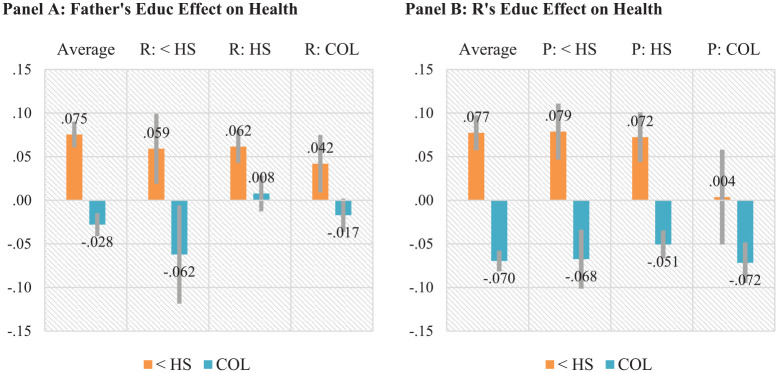
Average and Conditional Effects of Father’s and Own Education on Self-Reported Health, the Panel Study of Income Dynamics, 1968–2017. Panel A: Father’s Education Effect on Health. Panel B: Respondent’s Education Effect on Health. *Note:* The *y*-axis represents effects of completing college or having less than high school compared to completing high school on the probability of reporting poor or fair health. Figures in Panel A are average treatment effects and controlled direct effects of father’s education level from a weighted multilevel model with individual random intercepts (Equations 1 and 2). Figures in Panels B are average treatment effects of respondent’s own education level averaged over or conditional on father’s education from a weighted multilevel model with individual random intercepts (Equations 3 and 4). Gray bars indicate 95% confidence intervals. For additional significance results of second differences in each education effects, see Appendix Tables 1S and 2S in the online version of the article. <HS = less than high school; HS = high school completion; COL = college completion; R = respondent.

Figure 2 presents the estimated effects of father’s and own education on the likelihood of the respondent reporting poor or fair health; for numeric values, see Tables 1A and 2A in the online version of the article. Figure values are predicted probabilities of reporting poor or fair health with college completion and less than high school (<HS), respectively, compared to high school (HS) completion from Equations 1 to 4. Figure 2, Panel A depicts estimated ATEs and CDEs of father’s education on the respondent’s self-reported health after accounting for selection. The ATEs of father’s education suggest that greater father’s education reduces the likelihood of reporting poor or fair health. For example, after adjusting for selection, father’s college completion implies 2.8 percentage point (pp) lower probability (*p* < .01) of reporting poor or fair health, whereas father’s <HS education is associated with 7.5 pp (*p* < .01) higher probability of poor or fair health than those whose father completed HS. Such average effects of father’s education provide supporting evidence for Hypothesis 1a: Greater parental education—measured by father’s education—is causally linked to respondent’s better self-reported health.

The CDEs in Figure 2, Panel A assess whether the effects of father’s education on self-reported health differ in counterfactual populations where respondents (i.e., their children) complete <HS, HS, or college. It suggests that the parental educational gradients in health are larger in a counterfactual scenario in which respondents are less educated than a scenario in which respondents have greater education. For example, when all respondents hypothetically complete <HS, the educational difference between father’s college and <HS education in self-reported health is about 12.1 pp (5.9 to –(–6.2)), whereas the differences are 5.9 pp (4.2 –(–1.7)) and 5.4 pp (6.2 –.8) if respondents complete college and HS, respectively. These results provide empirical support for Hypothesis 4a that the influence of parental education is smaller when child generations have higher education. Additional significance results about the differences in these CDEs across scenarios are reported in Table 1S, top panel in the online version of the article.

Figure 2, Panel B reports the ATEs of own education and CATEs depending on father’s education level on self-reported health. The ATEs suggest that after considering selection, own <HS education implies 7.7 pp (*p* < .01) higher probability of reporting poor or fair health than HS completion, whereas college completion indicates 7.0 pp (*p* < .01) lower likelihood of reporting poor or fair health. The effect sizes of own education (i.e., the numeric values of the ATEs) are larger than that of father’s education. These ATEs of own education support Hypothesis 1a that own education has important implications for health.

The CATEs in Figure 2, Panel B assess Hypothesis 2 about whether the effect of own education on health depends on father’s education. Notably, the three CATEs of own college completion on self-reported health are comparable across the three levels of father’s education, indicating about 5.1 to 7.2 pp (all *p*s < .01) lower probability of reporting poor or fair health. These results support neither Hypotheses 2a nor 2b; rather, they suggest similar health benefits of college completion across educational backgrounds. At the same time, the negative effect of <HS appears to be larger for those whose fathers did not complete college (7.2 to 7.9 pp more likely, *p* < .01) than those whose fathers completed college (0.4 pp higher, *p* > .05). Additional significance results about the differences in these CATEs (see Table 2S, top panel in the online version of the article) provide consistent evidence.

**Figure 3.**
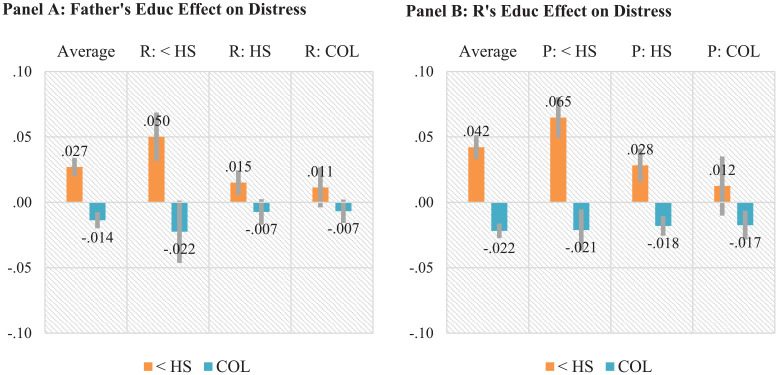
Marginal and Conditional Effects of Father’s and Own Education on Severe Psychological Distress, the Panel Study of Income Dynamics, 1968–2017. Panel A: Father’s Education Effect on Distress. Panel B: Respondent’s Education Effect on Distress. *Note:* The *y*-axis represents effects of completing college or having less than high school compared to completing high school on the probability of severe psychological distress. Figures in Panel A are average treatment effects and controlled direct effects of father’s level from a weighted multilevel model with individual random intercepts (Equations 1 and 2). Figures in Panel B are average treatment effects of respondent’s education level averaged over or conditional on father’s education from a weighted multilevel model with individual random intercepts (Equations 3 and 4). Gray bars indicate 95% confidence intervals. For additional significance results of second differences in each education effects, see Tables 1S and 2S in the online version of the article. <HS = less than high school; HS = high school completion; COL = college completion; R = respondent.

Figure 3 reports the average/marginal and conditional effects of parental and own education on severe psychological distress. Consistent with Hypothesis 1, the ATEs in Figure 1, Panel A suggest that father’s education has significant influence on the respondent’s psychological distress. Specifically, father’s college completion implies 1.4 pp (*p* < .001) lower probability of experiencing severe distress, whereas their <HS leads to a 2.7 pp (*p* < .001) higher probability than those whose parent completed HS.

In Figure 3, Panel A, the CDEs suggest that parental education would be more consequential in a counterfactual scenario where all respondents complete HS or less. Specifically, father’s <HS education would increase the probability of experiencing severe distress by 5.0 pp (*p* < .001) compared to those whose father completes HS. Father’s college completion implies 2.2 pp (*p* < .05) lower likelihood. In an alternative scenario in which all respondents complete college, father’s education does not seem to have a significant effect. Additional significance results about the differences in these CDEs (see Table 1S, bottom panel in the online version of the article) provide consistent evidence. This finding provides empirical evidence that is consistent with Hypothesis 4a that the influence of parental education may be smaller as the education level of the child generations increases.

Figure 3, Panel B reports the estimated ATEs and CATEs of own education on severe distress. After accounting for selection and pathways through various intermediate factors, <HS implies a 4.2 pp (*p* < .001) higher probability of experiencing severe distress than HS completion, whereas college completion indicates a 2.2 pp (*p* < .001) lower likelihood. The CATEs quantify the own education effects on severe distress for each of the three parental education groups. The three CATEs of college completion are largely similar across father’s education levels, indicating about 1.7 to 2.1 pp (all *p*s < .01) lower probability for respondents completing college than those who did not. The negative effect of <HS appears to be larger (6.5 pp, *p* < .001) if their father has <HS than those whose fathers had higher levels of education (2.8 pp higher, *p* < .001 and 1.2 pp higher, *p* > .05, respectively, associated with father’s HS and college completion). Additional significance results of differences in these CATEs (see Table 2S, bottom panel in the online version of the article) suggest consistent evidence.

**Figure 4.**
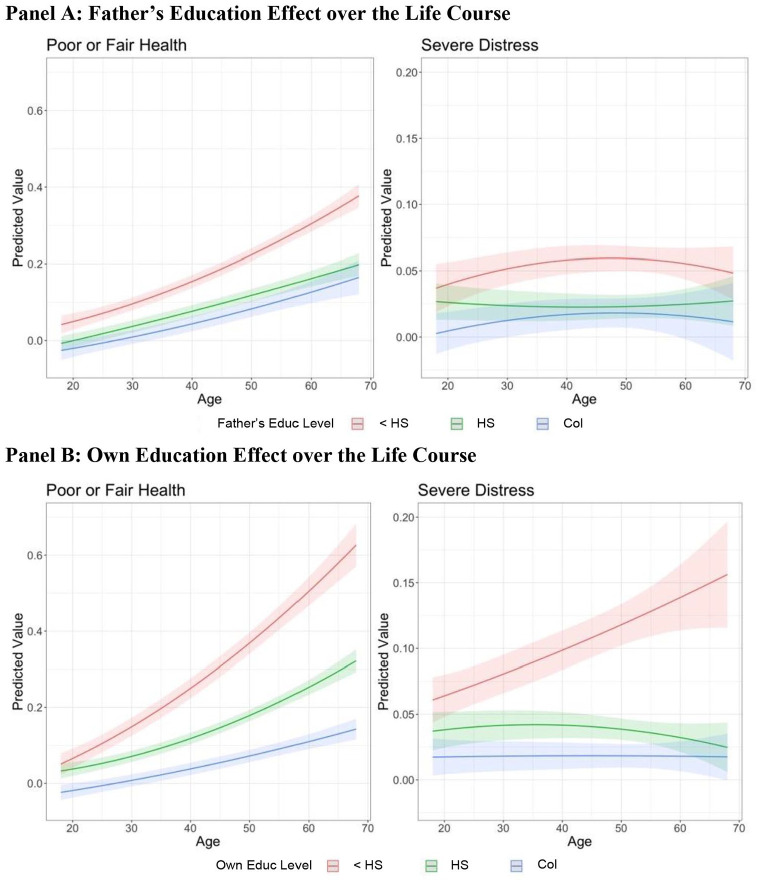
Father’s and Own Education Effects on Self-Reported Health and Severe Psychological Distress over the Life Course, the Panel Study of Income Dynamics, 1968–2017. Panel A: Father’s Education Effect over the Life Course. Panel B: Own Education Effect over the Life Course. *Note:* The *y*-axis represents predicted probabilities of reporting poor or fair health and severe distress by father’s (Panel A) and own (Panel B) education over the life course. Color bands are 95% confidence intervals. The predicted probabilities are based on a weighted multilevel model with a linear and a quadratic age term, cohort-specific fixed effect, and individual random intercepts. Educ = education; <HS = less than high school; HS = high school completion; Col = college completion.

The marginal and conditional effects reported in Figures 2 and 3 are averaged across the life course. Figure 4 depicts predicted probabilities of reporting poor or fair health and severe distress for each parental and own education group over the life course from the multilevel analyses. Figure 4, Panel A suggests that the life course changes of the parental education effects somewhat differ between the two outcomes. The probability of reporting poor or fair health increases with age for all parental education groups, whereas the likelihood of reporting psychological distress is highest in ages 40 and 50.

The differences among the three age-graded trajectories in Figure 4 indicate differences in parental educational gradients in each outcome over the life course. The parental educational gradients in severe psychological distress seem to be larger in midlife (i.e., ages 30–50) than at younger or older ages. For self-reported health, the parental education gradients appear to be relatively stable as the respondents age.

Figure 4, Panel B presents predicted probability of reporting poor or fair health and severe distress for three own education groups over the life course. The figure suggests that for all education groups, the likelihood of reporting poor or fair health increases with age, whereas the probability of experiencing psychological distress increases with age only for the lowest education group.

Figure 4 also suggests that the magnitude of the own educational gradients—indicated by the gaps between the three life course trajectories by education groups—varies across age groups and also between outcomes. Specifically, the own educational gradients in self-reported health seem to be larger as one gets older. By contrast, for severe distress, although the small effects of own college completion do not seem to vary over the life course, the negative effect of <HS appears to monotonically increase as one gets older, resulting in larger own educational gradients in older ages than in younger ages. Age-specific CATEs for the two health measures are available on request, displaying similar life course patterns as in the estimated ATEs in Figure 4.

All estimands are identifiable under the assumption that there are no important unobserved *X_P_* and *X_R_*. There may well be unobserved confounding variables not considered in the study. To assess the sensitivity of the findings to unobserved confounders, we conducted a series of nonparametric sensitivity analyses. The results suggest that the findings are largely robust to unobserved confounders in the sense that an unobserved confounder would be at least more than 10 times stronger than a common observed confounder to nullify the findings. Detailed discussions are provided in “Weighting Diagnostics and Sensitivity Analysis” in the Technical Appendix in the online version of the article.

## Discussion and Conclusions

Voluminous research has shown that both parent’s and respondent’s own education influence health. However, the question about for whom education conveys health benefits—that is, whether the health benefits of greater own education differ by parental education—was only examined in a handful of studies. Our research contributed to this emerging literature by explicating pathways and defining useful causal estimands in the education–health relationship in a two-generation setting. Using multilevel marginal structural models, we analyzed multigenerational data from the Panel Study of Income Dynamics—a nationally representative study that provides rich longitudinal data not only for the respondents but also for the parents. Our findings are twofold: First, at the individual level, college completion conveys similar health benefits across parental educational backgrounds. When the respondent has low education, lower parental education implies greater health risk. Second, at the population level, parent’s education may play a smaller role as the education level of the child’s generation increases.

Scholars have debated about if and the extent to which the education effect on health is causal and/or reflects the health selection. Prior research assessing the education effects on health largely relied on the respondent’s pre-education covariates. We took advantage of the multigenerational design of the PSID to better account for education and health selection not only for the respondents but also for their parents. Using MMSMs that consider a set of pre-education variables including childhood health conditions and family economic resources for both generations, we provide refined estimates of the health benefits of parent’s and own education. Our two-generational analysis generally supports Hypothesis 1a, the “social causation” hypothesis (Warren 2009; Warren et al. 2020), suggesting that greater parent’s and own education are likely causally linked to better health.

Importantly, our study finds that on average, the health benefits of completing college are similar regardless of their parent’s education levels, rejecting Hypothesis 2 that predicts differential health benefits by parental educational backgrounds. The health returns to college completion are also comparable for the two health measures. We note, however, this overall evidence of similar health benefits of education should not be taken to mean that education, either parent’s or own, influences health in the same way across parental educational backgrounds. For individuals of diverse socioeconomic backgrounds, the education–health link may operate through distinct pathways and mechanisms and/or in different directions. For example, social network disruption is generally thought to have negative health consequences. However, for those from a disadvantaged background, their mobility disrupts the old social networks and environment that expose the individuals to higher levels of health risk, such as smoking and violence, and simultaneously place them into a new network consisting of more health-oriented peers. Although the current analysis focuses on the average health benefits, future research should explicate the diverse forces that result in the similar overall effect of education on health across socioeconomic groups.

As the average education level increases in the United States, would the influence of parent’s education on adult children’s health increase or decrease? We utilize a well-defined causal quantity, the controlled direct effects of parental education, to assess this question. The estimated CDEs of parental education are smaller or even statistically nonsignificant in a hypothetical scenario where all respondents (i.e., children) complete high school or college than a scenario where respondents have less education. These results imply that consistent with Hypothesis 4a, parent’s education may be expected to play a smaller role in influencing adult children’s health in a population where the average educational level is higher. These suggestive findings are also consistent with the CATEs of own education discussed earlier; if greater own education implies better health for all socioeconomic groups, rising education levels among the younger generations are expected to overcome at least some of the negative influence of lower parental education.

We note, however, the conclusion about how parental education effects may change with the population educational compositions is based on CDE estimates from marginal structural models. It should be viewed as suggestive of a possible intervention relevance rather than definitive or predictive of future effects. Accurate predictions require a strong assumption that the health benefits of more education and the mechanisms are not a function of the average educational level in the population. Some education–health mechanisms may be sensitive to the educational compositions, which could imply differing education effects on health. For example, the relative education hypothesis posits that when college degrees are common, the social and economic returns could decrease due to more competition for limited high-paying occupations (Horowitz 2018), which, in turn, may reduce the health benefits of greater education. Other education–health mechanisms, such as knowledge and cognitive skills, may not differ depending on the educational composition in a population. We stress that our findings should be considered as initial evidence for an important question that needs to be addressed using additional data and perhaps different study designs.

Beyond confirming prior research showing life course patterns in the relationship between health and own education, our multilevel study provides new evidence about life course differences in parent’s education effects on two health measures. For example, the negative effects of own and parental education (i.e., not completing high school) on self-reported health appear to increase as one is older, providing support for the cumulative disadvantage hypothesis (Hypothesis 3). However, the effect of parental education on severe distress appears to be larger at ages 40 and 50 than younger or older ages. This curvilinear pattern is related to the diminishing effects of low parental education at age 60 or older, a life stage when parents may have passed away.

Our additional life course analyses (see Figure 4S in the online version of the article) suggest that the mixed findings in previous studies may be partly attributed to differing age distributions in the study samples. Based on samples of adult respondents age 20 to 25 or older, Bauldry (2014), Settels (2022), and Veenstra and Vanzella-Yang (2022) reported that a college degree was more beneficial for people from advantaged backgrounds. By contrast, Ross and Mirowsky (2011), Schaan (2014), and Vable et al. (2018) used data of age 60 or older respondents and found evidence supporting the recourse substitution hypothesis. As indicated by the gaps between education groups in Figure 1S in the online version of the article, greater education seems to provide a larger benefit in self-reported health for those born to better educated parents in ages 20 and 30. However, the benefits are larger for those born to less educated parents in older ages. It implies that the different age samples may contribute to the mixed findings reported in previous studies. This suggestive evidence, however, does not indicate that differing age distributions are the sole source underlying the seemingly conflicting results. Reconciling the mixed findings requires comprehensive and systematic meta-analysis of multiple data sources and analytical approaches, which is beyond the scope of the current study.

To measure parent’s education effects on health, we follow prior research to use father’s education in the main analysis. Researchers have increasingly noted the important role of mother’s education on health, although largely in low-income contexts or focusing on infant health or child development (Bender and McCann 2000; Karki Nepal 2018). We have thus considered an alternative measure of parental education using mother’s education in additional analyses and reported the results in Figures 1S to 3S in the online version of the article. The results about the influence of parental education on health and own education effect conditional on parental education are largely similar using father’s and mother’s education. The similar effects of father’s and mother’s education could reflect the assortative mating process in which partners match on educational attainment (Schwartz 2013) that subsequently consolidates socioeconomic resource and health behaviors (Maralani and Portier 2021). Empirically, these supplementary findings suggest that mother’s education may be a useful alternative measure of family background in studying the education–health link.

In this research, we focused on heterogeneity in the education–health relationship across two generations and treat race and gender as covariates. It has been shown that the health benefits of education differ by cohort, gender, race, and ethnicity (Andersson 2016; Daw et al. 2015; Umberson et al. 2014). We conducted additional analyses to explore potentially differing education–health associations between birth cohorts, men and women, and white and non-white samples in the PSID data. Tables 5S to 18S and Figure 5S in the online version of the article indicate that despite differences in numeric values, the main conclusions—that is, college completion conveys similar health benefits and parental education is more consequential when the average education level is low—are largely consistent across the subpopulations. Due to limited ethnicity information and sample size, we were unable to further examine ethnic differences within race groups. Future studies that examine the intersection of gender, racial, and ethnic heterogeneity using other data are merited.

The current study has limitations. First, we only considered two self-reported health outcomes. Because the mechanisms and pathways by which education influences health likely differ by health measures, it is possible that the effects of greater education are more pronounced in some outcomes and smaller in others. The empirical evidence about parent’s and own education effects on health is largely consistent between the two outcomes, but more research is needed to describe and explain potential heterogeneity in education effects on other health measures. Second, although we have included a set of parent- and respondent-level covariates shown to confound the education–health relationship, it is not possible to consider all confounders due to data availability. For example, recent studies using twin studies and natural experiments found that the education–health association was weakened by considering genetic and inheritable attributes (Behrman, Xiong, and Zhang 2015; Boardman and Fletcher 2015; Fujiwara and Kawachi 2009; Halpern-Manners et al. 2016), although there are possible spillover effects of sibling education and/or parental compensation on health (De Neve and Kawachi 2017). Unfortunately, although the PSID provides valuable life course data across generations, it lacks genetic information and offers only limited measures of early life cognitive abilities and noncognitive skills that are linked to educational attainment. Additional research is needed to address such confounding. Third, recent research suggested that some college, two-year college, and four-year college degrees have different health implications (Andersson, Wilkinson, and Maralani 2024; Zajacova et al. 2020). We are unable to distinguish these types of college education due to the limitation of the PSID’s education measures.

Despite these limitations, our study is among the first to consider a set of early life health-related factors for both the respondent and their parent generations for studying the education–health relationship. Our research provides a more rigorous assessment of the heterogeneous effects of education on health across generations. The similar health benefits of college education across socioeconomic backgrounds imply that increasing education may help overcome family disadvantages and offer health benefits for all socioeconomic groups. Our two-generation analysis shows that parental education effects on health are more pronounced in a scenario in which the child generation has a low level of education than if their educational level is high. This finding suggests that parental education might play a smaller role in influencing their children’s health if education levels continue to increase. Our study joins a growing recognition of increasing education as an effective strategy to improve population health and reduce health disparities.

## Supplemental Material

sj-docx-1-hsb-10.1177_00221465241249120 – Supplemental material for For Whom Does Education Convey Health Benefits? A Two-Generation and Life Course Approach Supplemental material, sj-docx-1-hsb-10.1177_00221465241249120 for For Whom Does Education Convey Health Benefits? A Two-Generation and Life Course Approach by Liying Luo and Lai Wei in Journal of Health and Social Behavior
