# Supplementary material for: For Whom Does Education Convey Health Benefits? A Two-Generation and Life Course Approach
Source: J Health Soc Behav. 2024 Jun 4;65(4):596–617. doi: 10.1177/00221465241249120 (PMC11622520; doi:10.1177/00221465241249120)
Supplement: sj-docx-1-hsb-10.1177_00221465241249120 – Supplemental material for For Whom Does Education Convey Health Benefits? A Two-Generation and Life Course Approach [file sj-docx-1-hsb-10.1177_00221465241249120.docx]

**Journal** of **Health**

and **Social Behavior**

OFFICIAL JOURNAL OF THE AMERICAN SOCIOLOGICAL ASSOCIATION

**ONLINE SUPPLEMENT**

**to article in**

Journal of Health and Social Behavior

**For Whom Does Education Convey Health Benefits? A Two-Generation and Life Course Approach**

**Liying Luo**

*The Pennsylvania State University*

**Lai Wei**

Princeton University


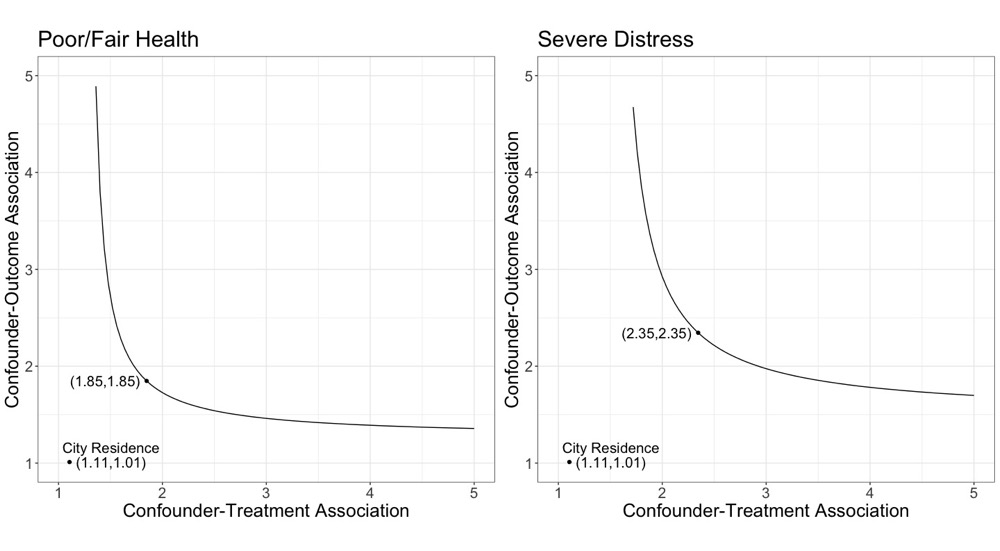


**Figure 1A. E-values and Sensitivity Curve of Father’s Education Effect to any Arbitrary Form of an Unobserved Confounder.**

*Note*: Treatment is father’s college completion. The curves indicate the confounding strength required to nullify the observed effect of father’s college completion on a health outcome. The confounder-treatment and confounder-outcome associations are measured on a risk ratio scale. The left panel shows that if an unobserved confounder could entirely account for the observed causal effect of father’s education on self-reported health, conditioning on all other observed confounders, the unobserved confounder would need to increase the likelihood of father’s college completion by 1.85 times, and also increases the probability of reporting good health status by 1.85 times. The right panel shows that if an unobserved confounder can entirely account for the observed causal effect of father’s college completion on severe distress, the unobserved confounder would need to increase the likelihood of father’s college completion by 2.35 times, and also increase the probability of experiencing severe distress by 2.34 times, conditioning on all other observed confounders.


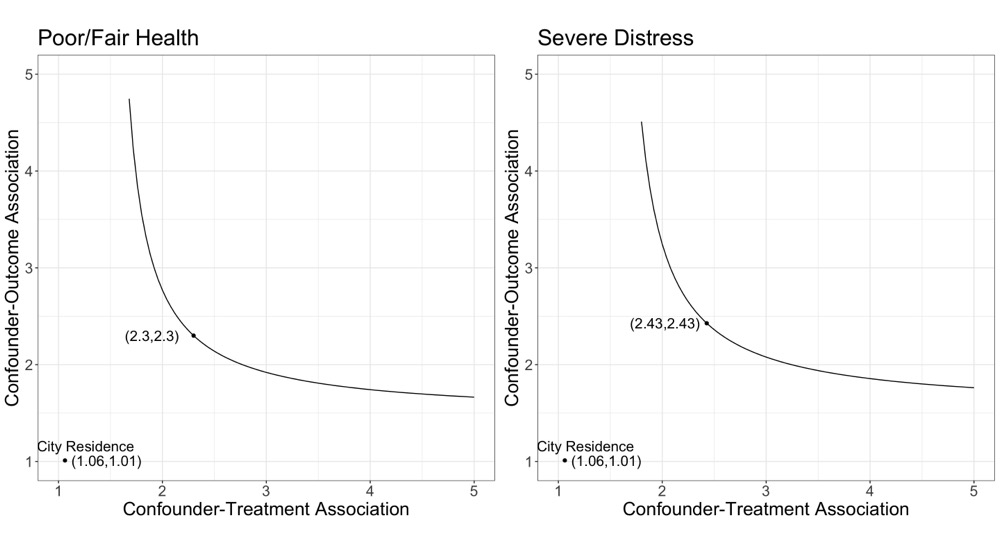


**Figure 2A. E-values and Sensitivity Curve of Own Education Effect to any Arbitrary Form of an Unobserved Confounder.**

*Note*: Treatment is own college completion. The curves indicate the confounding strength required to nullify the observed effect of own college completion on a health outcome. The confounder-treatment and confounder-outcome associations are measured on a risk ratio scale. The left panel shows that, if an unobserved confounder could entirely account for the observed causal effect of own education on self-reported health, conditioning on all other observed confounders, the unobserved confounder would need to increase the probability of completing college by 2.3 times, and increases the likelihood of reporting good health status by 2.3 times. The right panel shows that if an unobserved confounder could entirely account for the observed causal effect of own education on severe distress, the unobserved confounder would need to increase the likelihood of completing college by 2.43 times, and also increase the probability of experiencing severe distress by 2.43 times, conditioning on all other observed confounders.

**Figure 1S. Average and Conditional Effects of Mother’s and Own Education on Self-Reported Health, the Panel Study of Income Dynamics, 1968-2017.**

*Note*: Y-axis represents predicted probabilities of reporting poor or fair health. Figures in Panel A are average treatment effects and controlled direct effects of mother’s education from a weighted multilevel model with individual random intercepts. Figures in Panels B are average treatment effects of respondent’s own education averaged over or conditional on mother’s education from a weighted multilevel model with individual random intercepts. Grey bars indicate 95% confidence intervals. < HS: less than high school; HS: high school completion; COL: college completion.

**Figure 2S. Marginal and Conditional Effects of Mother’s and Own Education on Severe Psychological Distress, the Panel Study of Income Dynamics, 1968-2017.**

*Note*: Y-axis represents predicted probabilities of severe psychological distress. Figures in Panel A are average treatment effects and controlled direct effects of mother’s education from a weighted multilevel model with individual random intercepts. Figures in Panel B average treatment effects of respondent’s own education averaged over or conditional on mother’s education from a weighted multilevel model with individual random intercepts. Grey bars indicate 95% confidence intervals. < HS: less than high school; HS: high school completion; COL: college completion.


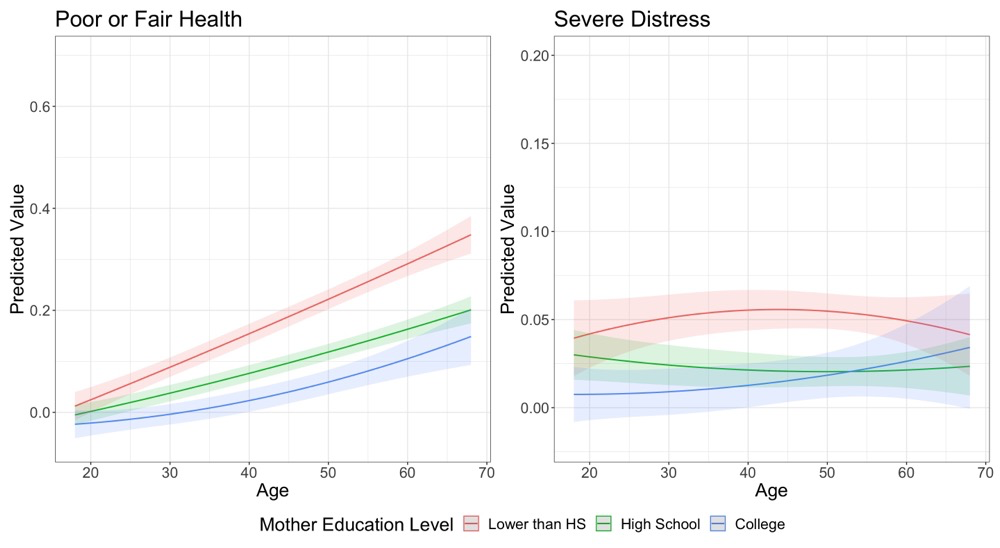


< HS

Mother’s Educ Level

HS

Col

**Figure 3S. Mother’s Education Effect on Self-Reported Health and Severe Psychological Distress over the Life Course, the Panel Study of Income Dynamics, 1968-2017.**

*Note*: Y-axis represents predicted probabilities of reporting poor or fair health and severe psychological distress by mother’s education over the life course. Color bands are 95% confidence intervals. The predicted probabilities are based on a weighted multilevel model with a linear and a quadratic age term, cohort-specific fixed effect, and individual random intercepts. < HS: less than high school; HS: high school completion; Col: college completion.


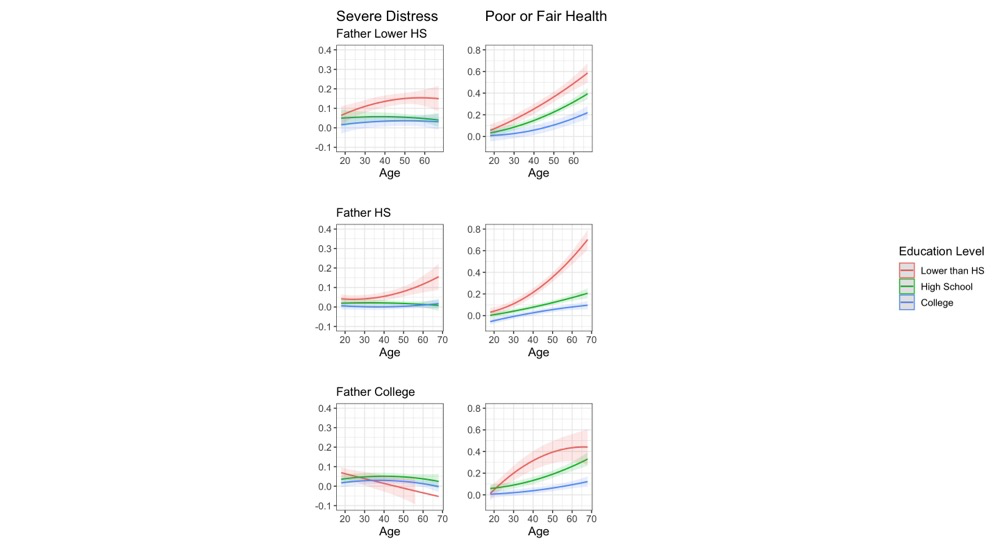


< HS

HS

Col

Own Educ Level


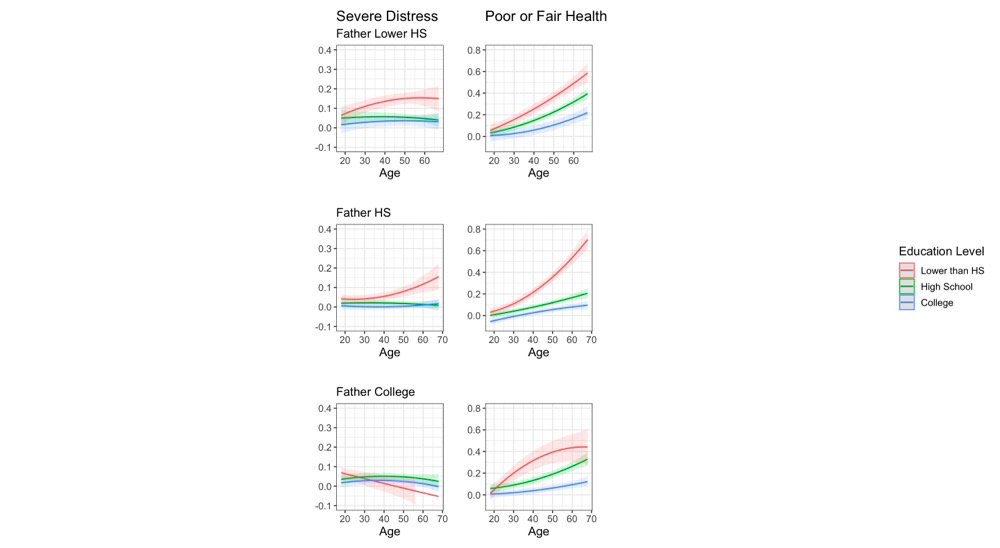


**Figure 4S. Own Education Effect by Father’s Education on Self-Reported Health and Severe Psychological Distress over the Life Course, the Panel Study of Income Dynamics, 1968-2017.**

*Note*: Y-axis represents predicted probabilities of reporting poor or fair health and severe distress by own and father’s education over the life course. Rows represent father’s education levels and columns different outcomes. Color bands are 95% confidence intervals. The predicted probabilities are based on a weighted multilevel model with linear and quadratic age terms, cohort-specific fixed effect, and individual random intercepts. < HS: less than high school; HS: high school completion; Col: college completion.

Panel A: Father’s Educ Effect over the Life Course


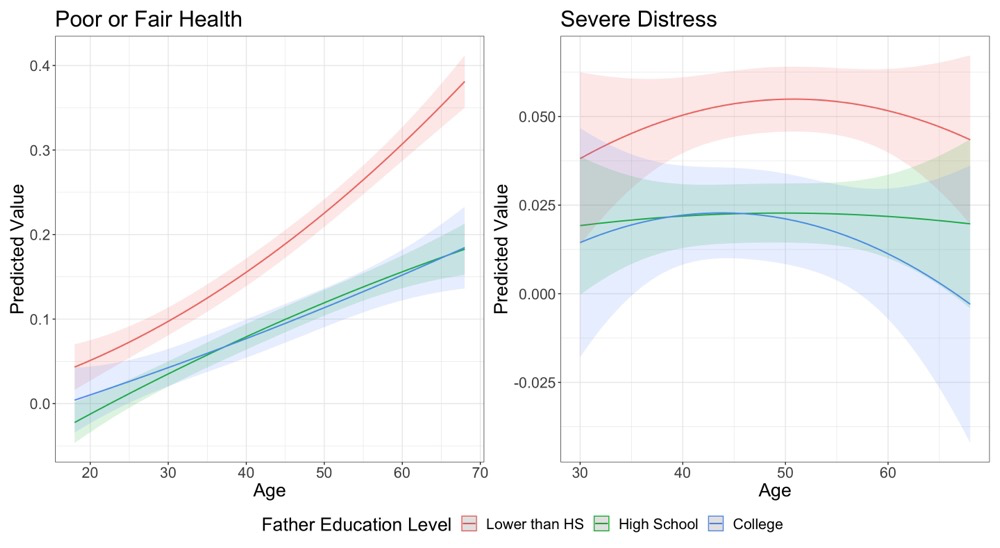


< HS

Father’s Educ Level

HS

Col

Panel B: Respondent’s Educ Effect over the Life Course


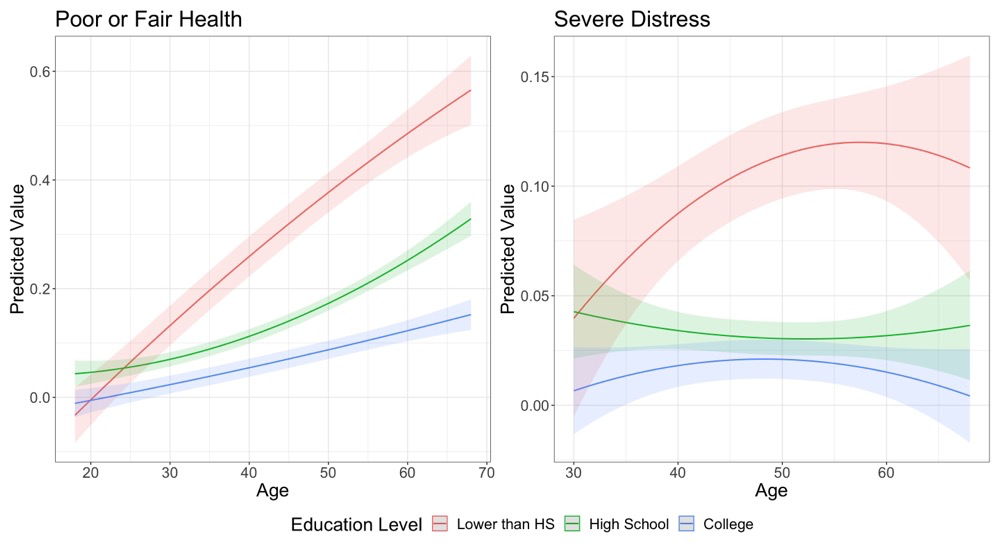


< HS

Own Educ Level

HS

Col

**Figure 5S. Father’s and Own Education Effects on Self-Reported Health and Severe Psychological Distress over the Life Course, the Panel Study of Income Dynamics: the 1950s and 1960s Cohort Sample.**

*Note*: Y-axis represents predicted probabilities of reporting poor or fair health and severe distress by father’s (Panel A) and respondent’s (Panel B) education over the life course. Color bands are 95% confidence intervals. The predicted probabilities are based on a weighted multilevel model with a linear and a quadratic age term, cohort-specific fixed effect, and individual random intercepts. < HS: less than high school; HS: high school completion; Col: college completion.

| **Table 1A. Marginal and Controlled Direct Effects of Father's Education on Health, the Panel Study of Income Dynamics, 1968-2017.** | | | | | | | | | | | | |  |  |
| --- | --- | --- | --- | --- | --- | --- | --- | --- | --- | --- | --- | --- | --- | --- |
|  |  |  |  |  |  |  |  |  |  |  |  |  |  |  |
|  |  |  |  |  |  |  |  |  |  |  |  |  |  |  |
| **Health Outcome** |  | **Educ Level** |  | **Marginal Effect** | |  | **Conditional Effect** | | | | | |  |  |
|  |  |  |  |  |  |  | **< HS** | | **HS** | | **Col** | |  |  |
|  |  |  |  |  |  |  |  |  |  |  |  |  |  |  |
|  |  |  |  |  |  |  |  |  |  |  |  |  |  |  |
| **Poor/Fair Health** |  | < HS |  | 0.075 | *** |  | 0.059 | *** | 0.062 | *** | 0.042 | ** |  |  |
|  |  |  |  | 0.008 |  |  | 0.020 |  | 0.009 |  | 0.017 |  |  |  |
|  |  | Col |  | -0.028 | *** |  | -0.062 | * | 0.008 |  | -0.017 | * |  |  |
|  |  |  |  | 0.007 |  |  | 0.029 |  | 0.011 |  | 0.010 |  |  |  |
|  |  |  |  |  |  |  |  |  |  |  |  |  |  |  |
|  |  |  |  |  |  |  |  |  |  |  |  |  |  |  |
| **Severe Distress** |  | < HS |  | 0.027 | *** |  | 0.050 | *** | 0.015 | ** | 0.011 |  |  |  |
|  |  |  |  | 0.004 |  |  | 0.009 |  | 0.005 |  | 0.008 |  |  |  |
|  |  | Col |  | -0.014 | *** |  | -0.022 | * | -0.007 |  | -0.007 |  |  |  |
|  |  |  |  | 0.003 |  |  | 0.012 |  | 0.005 |  | 0.005 |  |  |  |
|  |  |  |  |  |  |  |  |  |  |  |  |  |  |  |
|  |  |  |  |  |  |  |  |  |  |  |  |  |  |  |
| *Note*: Table figures represent probabilities. Table figures under "Marginal Effects" are average effects of father's education on each health measure estimated with weighted multilevel models. Table figures under "Conditional Effects" are controlled direct effect of father's education controlling for respondents' education. For general health, the number of unique father-child pairs = 6,405 and the total number of observations = 37,398. For psychological distress, the number of unique father-child pairs = 7,156 and the total number of observations = 39,725. High school completion is omitted as the reference group for all analyses. < HS: less than high school; Col: college completion. ***=p<0.001; **=p<0.01; *=p<0.05; two-tailed. | | | | | | | | | | | | | |  |
|  |  |  |  |  |  |  |  |  |  |  |  |  |  |  |

| **Table 2A. Marginal and Conditional Average Treatment Effects of Own Education on Health, the Panel Study of Income Dynamics, 1968-2017.** | | | | | | | | | | | | |  |
| --- | --- | --- | --- | --- | --- | --- | --- | --- | --- | --- | --- | --- | --- |
|  |  |  |  |  |  |  |  |  |  |  |  |  |  |
|  |  |  |  |  |  |  |  |  |  |  |  |  |  |
| **Health Outcome** |  | **Educ Level** |  | **Marginal Effect** | |  | **Conditional Effect** | | | | | |  |
|  |  |  |  |  |  |  | **< HS** | | **HS** | | **Col** | |  |
|  |  |  |  |  |  |  |  |  |  |  |  |  |  |
|  |  |  |  |  |  |  |  |  |  |  |  |  |  |
| **Poor/Fair Health** |  | < HS |  | 0.077 | *** |  | 0.079 | *** | 0.072 | *** | 0.004 |  |  |
|  |  |  |  | 0.010 |  |  | 0.016 |  | 0.015 |  | 0.028 |  |  |
|  |  | Col |  | -0.070 | *** |  | -0.068 | *** | -0.051 | *** | -0.072 | *** |  |
|  |  |  |  | 0.006 |  |  | 0.017 |  | 0.008 |  | 0.012 |  |  |
|  |  |  |  |  |  |  |  |  |  |  |  |  |  |
|  |  |  |  |  |  |  |  |  |  |  |  |  |  |
| **Severe Distress** |  | < HS |  | 0.042 | *** |  | 0.065 | *** | 0.028 | *** | 0.012 |  |  |
|  |  |  |  | 0.005 |  |  | 0.008 |  | 0.007 |  | 0.012 |  |  |
|  |  | Col |  | -0.022 | *** |  | -0.021 | ** | -0.018 | *** | -0.017 | ** |  |
|  |  |  |  | 0.003 |  |  | 0.008 |  | 0.004 |  | 0.006 |  |  |
|  |  |  |  |  |  |  |  |  |  |  |  |  |  |
|  |  |  |  |  |  |  |  |  |  |  |  |  |  |
| *Note*: Table figures represent probabilities. Table figures under "Marginal Effects" are average effects of father's education on each health measure estimated with weighted multilevel model. Table figures under "Conditional Effects" are conditional average treatment effects of respondents' education controlling for father's education. For general health, the number of unique father-child pairs = 6,405 and the total number of observations = 37,398. For psychological distress, the number of unique father-child pairs = 7,156 and the total number of observations = 39,725. High school completion is omitted as the reference group for all analyses. < HS: less than high school; Col: college completion. ***=p<0.001; **=p<0.01; *=p<0.05; two-tailed. | | | | | | | | | | | | |  |
|  |  |  |  |  |  |  |  |  |  |  |  |  |  |

| **Table 3A. Balance Check after CBPS Weighting for Estimating Father's and Own Education Effects on Health.** | | | | | | | | |
| --- | --- | --- | --- | --- | --- | --- | --- | --- |
|  |  |  |  |  |  |  |  |  |
|  |  |  |  |  |  |  |  |  |
| **Pre-Education Covariate** | | |  |  | **Maximum Standardized Difference** | | | |
|  |  |  |  |  |  |  |  |  |
|  |  |  |  |  | **For P's Educ Effect** |  | **For R's Educ Effect** | |
|  |  |  |  |  |  |  |  |  |
|  |  |  |  |  |  |  |  |  |
| **Name** |  | **Type** |  |  | **Father's** |  | **Father's** | **R's** |
|  |  |  |  |  |  |  |  |  |
| Race white |  | Binary |  |  | 0.01 |  | 0.01 | 0.02 |
| Race black |  | Binary |  |  | 0.00 |  | 0.01 | 0.02 |
| Race other |  | Binary |  |  | 0.03 |  | 0.02 | 0.01 |
| Adolescent depression |  | Binary |  |  | 0.02 |  | 0.02 | 0.02 |
| Adolescent substance |  | Binary |  |  | 0.03 |  | 0.03 | 0.02 |
| Adolescent health |  | Continuous |  |  | 0.01 |  | 0.01 | 0.02 |
| Adolescent emotion |  | Binary |  |  | 0.03 |  | 0.02 | 0.01 |
| Region Northeast |  | Binary |  |  | 0.01 |  | 0.01 | 0.04 |
| Region North |  | Binary |  |  | 0.02 |  | 0.00 | 0.01 |
| Region South |  | Binary |  |  | 0.01 |  | 0.01 | 0.00 |
| Region West |  | Binary |  |  | 0.01 |  | 0.02 | 0.02 |
| Residence farm |  | Binary |  |  | 0.02 |  | 0.02 | 0.01 |
| Residence suburb |  | Binary |  |  | 0.01 |  | 0.01 | 0.01 |
| Residence city |  | Binary |  |  | 0.01 |  | 0.02 | 0.01 |
| Residence other |  | Binary |  |  | 0.00 |  | 0.02 | 0.00 |
| Childhood parental SES |  | Continuous |  |  | 0.01 |  | 0.02 | 0.01 |
| Living with parents |  | Binary |  |  | 0.01 |  | 0.00 | 0.01 |
|  |  |  |  |  |  |  |  |  |
|  |  |  |  |  |  |  |  |  |

| *Note*: CBPS: covariate balancing propensity score. The estimation of parent's education effect uses fathers' characteristics. The estimation of own education effect uses both father's and the respondent's characteristics. Standardized difference is the difference in means divided by the variable's standard deviation. The maximum standardized difference for all possible pairs of educational level is reported. A conventional threshold to determine whether the covariates are well-balanced is 0.1 or less for standardized differences.   \| **Table 1S. Effect Differences in Controlled Direct Effects of Father's Education on Health by Respondent's Education, the Panel Study of Income Dynamics, 1968-2017.** \| \| \| \| \| \| \| \| \| \| \| \| \| --- \| --- \| --- \| --- \| --- \| --- \| --- \| --- \| --- \| --- \| --- \| --- \| \|  \|  \|  \|  \|  \|  \|  \|  \|  \|  \|  \|  \| \|  \|  \|  \|  \|  \|  \|  \|  \|  \|  \|  \|  \| \| **Health Outcome** \|  \| **Educ Level** \|  \| **Effect Difference** \| \| \| \| \| \| \| \| \|  \|  \| **< HS vs HS** \| \|  \| **Col vs HS** \| \|  \| **Col vs < HS** \| \| \|  \|  \|  \|  \|  \|  \|  \|  \|  \|  \|  \|  \| \|  \|  \|  \|  \|  \|  \|  \|  \|  \|  \|  \|  \| \| **Poor/Fair Health** \|  \| < HS \|  \| 0 \|  \|  \| -0.016 \|  \|  \| -0.016 \|  \| \|  \|  \|  \| (0.02) \|  \|  \| (0.018) \|  \|  \| (0.025) \|  \| \|  \| Col \|  \| 0.062 \| * \|  \| -0.025 \| * \|  \| 0.037 \|  \| \|  \|  \|  \|  \| (0.029) \|  \|  \| (0.013) \|  \|  \| (0.029) \|  \| \|  \|  \|  \|  \|  \|  \|  \|  \|  \|  \|  \|  \| \|  \|  \|  \|  \|  \|  \|  \|  \|  \|  \|  \|  \| \| **Severe Distress** \|  \| < HS \|  \| -0.035 \| *** \|  \| -0.004 \|  \|  \| -0.039 \| *** \| \|  \|  \|  \| (0.01) \|  \|  \| (0.009) \|  \|  \| (0.012) \|  \| \|  \| Col \|  \| 0.017 \|  \|  \| 0 \|  \|  \| 0.017 \|  \| \|  \|  \|  \|  \| (0.013) \|  \|  \| (0.007) \|  \|  \| (0.007) \|  \| \|  \|  \|  \|  \|  \|  \|  \|  \|  \|  \|  \|  \| \|  \|  \|  \|  \|  \|  \|  \|  \|  \|  \|  \|  \| \| *Note*: Table figures represent differences in controlled direct effect of father's education by respondents' education. For general health, the number of unique father-child pairs = 6,405 and the total number of observations = 37,398. For psychological distress, the number of unique father-child pairs = 7,156 and the total number of observations = 39,725. High school completion is omitted as the reference group for all analyses. < HS: less than high school; Col: college completion. ***=p<0.001; **=p<0.01; *=p<0.05; two-tailed. \| \| \| \| \| \| \| \| \| \| \| \| \|  \|  \| **Table 2S. Effect Differences in Conditional Average Treatment Effects of Respondent's Education on Health, the Panel Study of Income Dynamics, 1968-2017.** \| \| \| \| \| \| \| \| \| \| \| --- \| --- \| --- \| --- \| --- \| --- \| --- \| --- \| --- \| --- \| \|  \|  \|  \|  \|  \|  \|  \|  \|  \|  \| \|  \|  \|  \|  \|  \|  \|  \|  \|  \|  \| \| **Health Outcome** \|  \| **Educ Level** \|  \| **Effect Difference by R's Educ** \| \| \| \| \| \| \|  \|  \| **< HS vs HS** \| \| **Col vs HS** \| \| **Col vs < HS** \| \| \|  \|  \|  \|  \|  \|  \|  \|  \|  \|  \| \|  \|  \|  \|  \|  \|  \|  \|  \|  \|  \| \| **Poor/Fair Health** \|  \| < HS \|  \| -0.006 \|  \| -0.057 \| * \| -0.063 \| * \| \|  \|  \|  \| (0.02) \|  \| (0.03) \|  \| (0.031) \|  \| \|  \| Col \|  \| 0.015 \|  \| -0.022 \|  \| -0.007 \|  \| \|  \|  \|  \|  \| (0.017) \|  \| (0.013) \|  \| (0.019) \|  \| \|  \|  \|  \|  \|  \|  \|  \|  \|  \|  \| \|  \|  \|  \|  \|  \|  \|  \|  \|  \|  \| \| **Severe Distress** \|  \| < HS \|  \| -0.037 \| *** \| -0.016 \|  \| -0.053 \| *** \| \|  \|  \|  \| (0.01) \|  \| (0.013) \|  \| (0.014) \|  \| \|  \| Col \|  \| 0.003 \|  \| 0 \|  \| 0.003 \|  \| \|  \|  \|  \|  \| (0.008) \|  \| (0.007) \|  \| (0.01) \|  \| \|  \|  \|  \|  \|  \|  \|  \|  \|  \|  \| \|  \|  \|  \|  \|  \|  \|  \|  \|  \|  \| \| *Note*: Table figures represent differences in conditional average treatment effect of respondent's education by father' education. For general health, the number of unique father-child pairs = 6,405 and the total number of observations = 37,398. For psychological distress, the number of unique father-child pairs = 7,156 and the total number of observations = 39,725. High school completion is omitted as the reference group for all analyses. < HS: less than high school; Col: college completion. ***=p<0.001; **=p<0.01; *=p<0.05; two-tailed. \| \| \| \| \| \| \| \| \| \| \|  \|  \| **Table 3S. Marginal and Controlled Direct Effects of Father's Education on Health without Imputing Controls.** \| \| \| \| \| \| \| \| \| \| \| \| \|  \| \| --- \| --- \| --- \| --- \| --- \| --- \| --- \| --- \| --- \| --- \| --- \| --- \| --- \| --- \| \|  \|  \|  \|  \|  \|  \|  \|  \|  \|  \|  \|  \|  \|  \| \|  \|  \|  \|  \|  \|  \|  \|  \|  \|  \|  \|  \|  \|  \| \| **Health Outcome** \|  \| **Educ Level** \|  \| **Marginal Effect** \| \|  \| **Conditional Effect** \| \| \| \| \| \|  \| \|  \|  \|  \| **< HS** \| \| **HS** \| \| **Col** \| \|  \| \|  \|  \|  \|  \|  \|  \|  \|  \|  \|  \|  \|  \|  \|  \| \|  \|  \|  \|  \|  \|  \|  \|  \|  \|  \|  \|  \|  \|  \| \| **Poor/Fair Health** \|  \| < HS \|  \| 0.030 \| * \|  \| -0.011 \|  \| 0.021 \|  \| 0.014 \|  \|  \| \|  \|  \|  \| 0.014 \|  \|  \| 0.036 \|  \| 0.018 \|  \| 0.028 \|  \|  \| \|  \| Col \|  \| -0.031 \| ** \|  \| -0.100 \|  \| -0.012 \|  \| -0.015 \|  \|  \| \|  \|  \|  \|  \| 0.011 \|  \|  \| 0.072 \|  \| 0.017 \|  \| 0.016 \|  \|  \| \|  \|  \|  \|  \|  \|  \|  \|  \|  \|  \|  \|  \|  \|  \| \|  \|  \|  \|  \|  \|  \|  \|  \|  \|  \|  \|  \|  \|  \| \| **Severe Distress** \|  \| < HS \|  \| 0.012 \|  \|  \| 0.026 \|  \| -0.003 \|  \| 0.010 \|  \|  \| \|  \|  \|  \| 0.007 \|  \|  \| 0.019 \|  \| 0.010 \|  \| 0.015 \|  \|  \| \|  \| Col \|  \| -0.009 \|  \|  \| -0.035 \|  \| 0.007 \|  \| -0.011 \|  \|  \| \|  \|  \|  \|  \| 0.006 \|  \|  \| 0.039 \|  \| 0.009 \|  \| 0.008 \|  \|  \| \|  \|  \|  \|  \|  \|  \|  \|  \|  \|  \|  \|  \|  \|  \| \|  \|  \|  \|  \|  \|  \|  \|  \|  \|  \|  \|  \|  \|  \| \| *Note*: Table figures represent probabilities. Table figures under "Marginal Effects" are average effects of father's education on each health measure estimated with weighted multilevel models. Table figures under "Conditional Effects" are controlled direct effect of father's education controlling for respondents' education. For general health, the number of unique father-child pairs = 1,957 and the total number of observations = 10,751. For psychological distress, the number of unique father-child pairs = 1,971 and the total number of observations = 12,946. High school graduate is omitted as the reference group for all outcomes. < HS: less than high school; Col: college completion. ***=p<0.001; **=p<0.01; *=p<0.05; two-tailed. \| \| \| \| \| \| \| \| \| \| \| \| \| \| \|  \|  \| **Table 4S. Marginal and Conditional Average Treatment Effects of Respondent's Education on Health without Imputing Controls.** \| \| \| \| \| \| \| \| \| \| \| \| \| \| --- \| --- \| --- \| --- \| --- \| --- \| --- \| --- \| --- \| --- \| --- \| --- \| --- \| \|  \|  \|  \|  \|  \|  \|  \|  \|  \|  \|  \|  \|  \| \|  \|  \|  \|  \|  \|  \|  \|  \|  \|  \|  \|  \|  \| \| **Health Outcome** \|  \| **Educ Level** \|  \| **Marginal Effect** \| \|  \| **Conditional Effect** \| \| \| \| \| \| \|  \|  \|  \| **< HS** \| \| **HS** \| \| **Col** \| \| \|  \|  \|  \|  \|  \|  \|  \|  \|  \|  \|  \|  \|  \| \|  \|  \|  \|  \|  \|  \|  \|  \|  \|  \|  \|  \|  \| \| **Poor/Fair Health** \|  \| < HS \|  \| 0.093 \| *** \|  \| 0.076 \| * \| 0.100 \| *** \| 0.022 \|  \| \|  \|  \|  \| 0.018 \|  \|  \| 0.033 \|  \| 0.024 \|  \| 0.068 \|  \| \|  \| Col \|  \| -0.055 \| *** \|  \| -0.048 \|  \| -0.051 \| *** \| -0.048 \| ** \| \|  \|  \|  \|  \| 0.009 \|  \|  \| 0.033 \|  \| 0.013 \|  \| 0.017 \|  \| \|  \|  \|  \|  \|  \|  \|  \|  \|  \|  \|  \|  \|  \| \|  \|  \|  \|  \|  \|  \|  \|  \|  \|  \|  \|  \|  \| \| **Severe Distress** \|  \| < HS \|  \| 0.043 \| *** \|  \| 0.059 \| ** \| 0.042 \| ** \| -0.003 \|  \| \|  \|  \|  \| 0.010 \|  \|  \| 0.018 \|  \| 0.013 \|  \| 0.036 \|  \| \|  \| Col \|  \| -0.018 \| *** \|  \| 0.003 \|  \| -0.013 \| * \| -0.030 \| *** \| \|  \|  \|  \|  \| 0.005 \|  \|  \| 0.017 \|  \| 0.006 \|  \| 0.009 \|  \| \|  \|  \|  \|  \|  \|  \|  \|  \|  \|  \|  \|  \|  \| \|  \|  \|  \|  \|  \|  \|  \|  \|  \|  \|  \|  \|  \| \| *Note*: Table figures represent probabilities. Table figures under "Marginal Effects" are average effects of respondent's own education on each health measure estimated with weighted multilevel model. Table figures under "Conditional Effects" are conditional average treatment effects of respondents' education controlling for father's education. For general health, the number of unique father-child pairs = 1,957 and the total number of observations = 10,751. For psychological distress, the number of unique father-child pairs = 1,971 and the total number of observations = 12,946. High school graduate is omitted as the reference group for all outcomes. < HS: less than high school; Col: college completion. ***=p<0.001; **=p<0.01; *=p<0.05; two-tailed. \| \| \| \| \| \| \| \| \| \| \| \| \| \|  \|  \| **Table 5S. Marginal and Controlled Direct Effects of Father's Education on Alternative Health Measures, the Panel Study of Income Dynamics, 1968-2017.** \| \| \| \| \| \| \| \| \| \| \| \| \|  \| \| --- \| --- \| --- \| --- \| --- \| --- \| --- \| --- \| --- \| --- \| --- \| --- \| --- \| --- \| \|  \|  \|  \|  \|  \|  \|  \|  \|  \|  \|  \|  \|  \|  \| \|  \|  \|  \|  \|  \|  \|  \|  \|  \|  \|  \|  \|  \|  \| \| **Health Outcome** \|  \| **Educ Level** \|  \| **Marginal Effect** \| \|  \| **Conditional Effect** \| \| \| \| \| \|  \| \|  \|  \|  \| **< HS** \| \| **HS** \| \| **Col** \| \|  \| \|  \|  \|  \|  \|  \|  \|  \|  \|  \|  \|  \|  \|  \|  \| \|  \|  \|  \|  \|  \|  \|  \|  \|  \|  \|  \|  \|  \|  \| \| **Self-Rated Health** \|  \| < HS \|  \| 0.301 \| *** \|  \| 0.207 \| *** \| 0.3 \| *** \| 0.203 \| *** \|  \| \|  \|  \|  \| 0.011 \|  \|  \| 0.027 \|  \| 0.017 \|  \| 0.016 \|  \|  \| \|  \| Col \|  \| -0.052 \| *** \|  \| 0.107 \|  \| 0.033 \|  \| -0.104 \|  \|  \| \|  \|  \|  \| 0.012 \|  \|  \| 0.037 \|  \| 0.019 \|  \| 0.016 \|  \|  \| \|  \|  \|  \|  \|  \|  \|  \|  \|  \|  \|  \|  \|  \|  \| \|  \|  \|  \|  \|  \|  \|  \|  \|  \|  \|  \|  \|  \|  \| \| **K-6 Sore** \|  \| < HS \|  \| 0.69 \| *** \|  \| 0.915 \| *** \| 0.572 \| *** \| 0.051 \|  \|  \| \|  \|  \|  \| 0.05 \|  \|  \| 0.121 \|  \| 0.079 \|  \| 0.071 \|  \|  \| \|  \| Col \|  \| -0.3 \| *** \|  \| -0.765 \| *** \| -0.155 \| * \| -0.117 \|  \|  \| \|  \|  \|  \| 0.044 \|  \|  \| 0.134 \|  \| 0.075 \|  \| 0.063 \|  \|  \| \|  \|  \|  \|  \|  \|  \|  \|  \|  \|  \|  \|  \|  \|  \| \|  \|  \|  \|  \|  \|  \|  \|  \|  \|  \|  \|  \|  \|  \| \| *Note*: Table figures represent probabilities. Table figures under "Marginal Effects" are average effects of father's education on each health measure estimated with weighted multilevel models. Table figures under "Conditional Effects" are controlled direct effect of father's education controlling for respondents' education. For general health, the number of unique father-child pairs = 6,405 and the total number of observations = 37,398. For psychological distress, the number of unique father-child pairs = 7,156 and the total number of observations = 39,725. High school graduate is omitted as the reference group for all outcomes. < HS: less than high school; Col: college completion. ***=p<0.001; **=p<0.01; *=p<0.05; two-tailed. \| \| \| \| \| \| \| \| \| \| \| \| \| \| \|  \|  \| **Table 6S. Marginal and Conditional Average Treatment Effects of Respondent's Education on Alternative Health Measures, the Panel Study of Income Dynamics, 1968-2017.** \| \| \| \| \| \| \| \| \| \| \| \| \| \| --- \| --- \| --- \| --- \| --- \| --- \| --- \| --- \| --- \| --- \| --- \| --- \| --- \| \|  \|  \|  \|  \|  \|  \|  \|  \|  \|  \|  \|  \|  \| \|  \|  \|  \|  \|  \|  \|  \|  \|  \|  \|  \|  \|  \| \| **Health Outcome** \|  \| **Educ Level** \|  \| **Marginal Effect** \| \|  \| **Conditional Effect** \| \| \| \| \| \| \|  \|  \|  \| **< HS** \| \| **HS** \| \| **Col** \| \| \|  \|  \|  \|  \|  \|  \|  \|  \|  \|  \|  \|  \|  \| \|  \|  \|  \|  \|  \|  \|  \|  \|  \|  \|  \|  \|  \| \| **Self-Rated Health** \|  \| < HS \|  \| 0.328 \| *** \|  \| 0.235 \| *** \| 0.308 \| *** \| 0.312 \| *** \| \|  \|  \|  \| 0.014 \|  \|  \| 0.02 \|  \| 0.021 \|  \| 0.045 \|  \| \|  \| Col \|  \| -0.286 \| *** \|  \| -0.265 \| *** \| -0.23 \| *** \| -0.359 \| *** \| \|  \|  \|  \| 0.01 \|  \|  \| 0.018 \|  \| 0.013 \|  \| 0.022 \|  \| \|  \|  \|  \|  \|  \|  \|  \|  \|  \|  \|  \|  \|  \| \|  \|  \|  \|  \|  \|  \|  \|  \|  \|  \|  \|  \|  \| \| **K-6 Sore** \|  \| < HS \|  \| 1.11 \| *** \|  \| 1.39 \| *** \| 1.072 \| *** \| 0.001 \|  \| \|  \|  \|  \| 0.061 \|  \|  \| 0.109 \|  \| 0.084 \|  \| 0.148 \|  \| \|  \| Col \|  \| -0.617 \| *** \|  \| -1.011 \| *** \| -0.516 \| *** \| -0.407 \| *** \| \|  \|  \|  \| 0.041 \|  \|  \| 0.089 \|  \| 0.054 \|  \| 0.087 \|  \| \|  \|  \|  \|  \|  \|  \|  \|  \|  \|  \|  \|  \|  \| \|  \|  \|  \|  \|  \|  \|  \|  \|  \|  \|  \|  \|  \| \| *Note*: Table figures represent probabilities. Table figures under "Marginal Effects" are average effects of father's education on each health measure estimated with weighted multilevel model. Table figures under "Conditional Effects" are conditional average treatment effects of respondents' education controlling for father's education. For general health, the number of unique father-child pairs = 6,405 and the total number of observations = 37,398. For psychological distress, the number of unique father-child pairs = 7,156 and the total number of observations = 39,725. High school graduate is omitted as the reference group for all outcomes. < HS: less than high school; Col: college completion. ***=p<0.001; **=p<0.01; *=p<0.05; two-tailed. \| \| \| \| \| \| \| \| \| \| \| \| \| \|  \|  \| **Table 7S. Marginal and Controlled Direct Effects of Father's Education on Health: Men Sample.** \| \| \| \| \| \| \| \| \| \| \| \| \|  \| \| --- \| --- \| --- \| --- \| --- \| --- \| --- \| --- \| --- \| --- \| --- \| --- \| --- \| --- \| \|  \|  \|  \|  \|  \|  \|  \|  \|  \|  \|  \|  \|  \|  \| \|  \|  \|  \|  \|  \|  \|  \|  \|  \|  \|  \|  \|  \|  \| \| **Health Outcome** \|  \| **Educ Level** \|  \| **Marginal Effect** \| \|  \| **Conditional Effect** \| \| \| \| \| \|  \| \|  \|  \|  \| **< HS** \| \| **HS** \| \| **Col** \| \|  \| \|  \|  \|  \|  \|  \|  \|  \|  \|  \|  \|  \|  \|  \|  \| \|  \|  \|  \|  \|  \|  \|  \|  \|  \|  \|  \|  \|  \|  \| \| **Poor/Fair Health** \|  \| < HS \|  \| 0.069 \| *** \|  \| 0.042 \| * \| 0.056 \| *** \| 0.024 \|  \|  \| \|  \|  \|  \| 0.009 \|  \|  \| 0.019 \|  \| 0.010 \|  \| 0.020 \|  \|  \| \|  \| Col \|  \| -0.028 \| ** \|  \| -0.064 \| * \| 0.008 \|  \| -0.011 \|  \|  \| \|  \|  \|  \|  \| 0.008 \|  \|  \| 0.031 \|  \| 0.011 \|  \| 0.011 \|  \|  \| \|  \|  \|  \|  \|  \|  \|  \|  \|  \|  \|  \|  \|  \|  \| \|  \|  \|  \|  \|  \|  \|  \|  \|  \|  \|  \|  \|  \|  \| \| **Severe Distress** \|  \| < HS \|  \| 0.031 \| *** \|  \| 0.042 \| *** \| 0.016 \| *** \| 0.031 \| ** \|  \| \|  \|  \|  \| 0.005 \|  \|  \| 0.012 \|  \| 0.006 \|  \| 0.012 \|  \|  \| \|  \| Col \|  \| -0.009 \| * \|  \| -0.042 \| ** \| 0.000 \|  \| 0.000 \|  \|  \| \|  \|  \|  \|  \| 0.004 \|  \|  \| 0.015 \|  \| 0.006 \|  \| 0.006 \|  \|  \| \|  \|  \|  \|  \|  \|  \|  \|  \|  \|  \|  \|  \|  \|  \| \|  \|  \|  \|  \|  \|  \|  \|  \|  \|  \|  \|  \|  \|  \| \| *Note*: Table figures represent probabilities. Table figures under "Marginal Effects" are marginal effects of father's education on each health measure estimated with weighted multilevel models. Table figures under "Conditional Effects" are controlled direct effect of father's education controlling for respondents' education. For general health, the number of unique father-child pairs = 3,219 and the total number of observations = 24,355. For psychological distress, the number of unique father-child pairs = 3,585 and the total number of observations = 19,198. High school graduate is omitted as the reference group for all outcomes. < HS: less than high school; Col: college completion. ***=p<0.001 ; **=p<0.01 ; *=p<0.05; two-tailed. \| \| \| \| \| \| \| \| \| \| \| \| \| \| \|  \|  \| **Table 8S. Marginal and Controlled Direct Effects of Father's Education on Health: Women Sample.** \| \| \| \| \| \| \| \| \| \| \| \| \|  \| \| --- \| --- \| --- \| --- \| --- \| --- \| --- \| --- \| --- \| --- \| --- \| --- \| --- \| --- \| \|  \|  \|  \|  \|  \|  \|  \|  \|  \|  \|  \|  \|  \|  \| \|  \|  \|  \|  \|  \|  \|  \|  \|  \|  \|  \|  \|  \|  \| \| **Health Outcome** \|  \| **Educ Level** \|  \| **Marginal Effect** \| \|  \| **Conditional Effect** \| \| \| \| \| \|  \| \|  \|  \|  \| **< HS** \| \| **HS** \| \| **Col** \| \|  \| \|  \|  \|  \|  \|  \|  \|  \|  \|  \|  \|  \|  \|  \|  \| \|  \|  \|  \|  \|  \|  \|  \|  \|  \|  \|  \|  \|  \|  \| \| **Poor/Fair Health** \|  \| < HS \|  \| 0.076 \| *** \|  \| 0.133 \| *** \| 0.062 \| *** \| 0.062 \| ** \|  \| \|  \|  \|  \| 0.012 \|  \|  \| 0.029 \|  \| 0.013 \|  \| 0.021 \|  \|  \| \|  \| Col \|  \| -0.026 \|  \|  \| -0.025 \|  \| 0.013 \|  \| -0.019 \|  \|  \| \|  \|  \|  \|  \| 0.011 \|  \|  \| 0.044 \|  \| 0.017 \|  \| 0.015 \|  \|  \| \|  \|  \|  \|  \|  \|  \|  \|  \|  \|  \|  \|  \|  \|  \| \|  \|  \|  \|  \|  \|  \|  \|  \|  \|  \|  \|  \|  \|  \| \| **Severe Distress** \|  \| < HS \|  \| 0.022 \| *** \|  \| 0.062 \| *** \| 0.012 \|  \| 0.002 \|  \|  \| \|  \|  \|  \| 0.005 \|  \|  \| 0.015 \|  \| 0.007 \|  \| 0.010 \|  \|  \| \|  \| Col \|  \| -0.019 \| ** \|  \| -0.007 \|  \| -0.016 \| * \| -0.012 \| * \|  \| \|  \|  \|  \|  \| 0.005 \|  \|  \| 0.019 \|  \| 0.008 \|  \| 0.007 \|  \|  \| \|  \|  \|  \|  \|  \|  \|  \|  \|  \|  \|  \|  \|  \|  \| \|  \|  \|  \|  \|  \|  \|  \|  \|  \|  \|  \|  \|  \|  \| \| *Note*: Table figures represent probabilities. Table figures under "Marginal Effects" are marginal effects of father's education on each health measure estimated with weighted multilevel models. Table figures under "Conditional Effects" are controlled direct effect of father's education controlling for respondents' education. For general health, the number of unique father-child pairs = 3,186 and the total number of observations = 13,043. For psychological distress, the number of unique father-child pairs = 3,571 and the total number of observations = 20,527. < HS: less than high school; Col: college completion. ***=p<0.001 ; **=p<0.01 ; *=p<0.05; two-tailed. \| \| \| \| \| \| \| \| \| \| \| \| \| \| \|  \|  \| **Table 9S. Marginal and Conditional Average Treatment Effects of Respondent's Education on Health: Men Sample.** \| \| \| \| \| \| \| \| \| \| \| \| \| \| --- \| --- \| --- \| --- \| --- \| --- \| --- \| --- \| --- \| --- \| --- \| --- \| --- \| \|  \|  \|  \|  \|  \|  \|  \|  \|  \|  \|  \|  \|  \| \|  \|  \|  \|  \|  \|  \|  \|  \|  \|  \|  \|  \|  \| \| **Health Outcome** \|  \| **Educ Level** \|  \| **Marginal Effect** \| \|  \| **Conditional Effect** \| \| \| \| \| \| \|  \|  \|  \| **< HS** \| \| **HS** \| \| **Col** \| \| \|  \|  \|  \|  \|  \|  \|  \|  \|  \|  \|  \|  \|  \| \|  \|  \|  \|  \|  \|  \|  \|  \|  \|  \|  \|  \|  \| \| **Poor/Fair Health** \|  \| < HS \|  \| 0.075 \| ******* \|  \| 0.067 \| *** \| 0.076 \| *** \| 0.006 \|  \| \|  \|  \|  \| 0.010 \|  \|  \| 0.015 \|  \| 0.015 \|  \| 0.031 \|  \| \|  \| Col \|  \| -0.070 \| *** \|  \| -0.081 \| *** \| -0.052 \| *** \| -0.068 \| *** \| \|  \|  \|  \|  \| 0.007 \|  \|  \| 0.020 \|  \| 0.010 \|  \| 0.013 \|  \| \|  \|  \|  \|  \|  \|  \|  \|  \|  \|  \|  \|  \|  \| \|  \|  \|  \|  \|  \|  \|  \|  \|  \|  \|  \|  \|  \| \| **Severe Distress** \|  \| < HS \|  \| 0.040 \| *** \|  \| 0.060 \| *** \| 0.035 \| *** \| -0.007 \|  \| \|  \|  \|  \| 0.006 \|  \|  \| 0.011 \|  \| 0.008 \|  \| 0.015 \|  \| \|  \| Col \|  \| -0.019 \| *** \|  \| -0.003 \|  \| -0.017 \| *** \| -0.017 \| * \| \|  \|  \|  \|  \| 0.004 \|  \|  \| 0.012 \|  \| 0.005 \|  \| 0.007 \|  \| \|  \|  \|  \|  \|  \|  \|  \|  \|  \|  \|  \|  \|  \| \|  \|  \|  \|  \|  \|  \|  \|  \|  \|  \|  \|  \|  \| \| *Note*: Table figures represent probabilities. Table figures under "Marginal Effects" are marginal effects of respondent's education on each health measure estimated with weighted multilevel model. Table figures under "Conditional Effects" are conditional average treatment effects of respondents' education controlling for father's education. For general health, the number of unique father-child pairs = 3,219 and the total number of observations = 24,355. For psychological distress, the number of unique father-child pairs = 3,585 and the total number of observations = 19,198. High school graduate is omitted as the reference group for all outcomes. < HS: less than high school; Col: college completion. ***=p<0.001 ; **=p<0.01 ; *=p<0.05; two-tailed. \| \| \| \| \| \| \| \| \| \| \| \| \| \|  \|  \| **Table 10S. Marginal and Conditional Average Treatment Effects of Respondent's Education on Health: Women Sample.** \| \| \| \| \| \| \| \| \| \| \| \| \| \| --- \| --- \| --- \| --- \| --- \| --- \| --- \| --- \| --- \| --- \| --- \| --- \| --- \| \|  \|  \|  \|  \|  \|  \|  \|  \|  \|  \|  \|  \|  \| \|  \|  \|  \|  \|  \|  \|  \|  \|  \|  \|  \|  \|  \| \| **Health Outcome** \|  \| **Educ Level** \|  \| **Marginal Effect** \| \|  \| **Conditional Effect** \| \| \| \| \| \| \|  \|  \|  \| **< HS** \| \| **HS** \| \| **Col** \| \| \|  \|  \|  \|  \|  \|  \|  \|  \|  \|  \|  \|  \|  \| \|  \|  \|  \|  \|  \|  \|  \|  \|  \|  \|  \|  \|  \| \| **Poor/Fair Health** \|  \| < HS \|  \| 0.121 \| *** \|  \| 0.149 \| *** \| 0.075 \| ** \| 0.044 \|  \| \|  \|  \|  \| 0.014 \|  \|  \| 0.020 \|  \| 0.023 \|  \| 0.042 \|  \| \|  \| Col \|  \| -0.056 \| *** \|  \| -0.038 \|  \| -0.038 \| ** \| -0.066 \| *** \| \|  \|  \|  \|  \| 0.009 \|  \|  \| 0.021 \|  \| 0.012 \|  \| 0.019 \|  \| \|  \|  \|  \|  \|  \|  \|  \|  \|  \|  \|  \|  \|  \| \|  \|  \|  \|  \|  \|  \|  \|  \|  \|  \|  \|  \|  \| \| **Severe Distress** \|  \| < HS \|  \| 0.046 \| *** \|  \| 0.072 \| *** \| 0.019 \|  \| 0.029 \|  \| \|  \|  \|  \| 0.007 \|  \|  \| 0.012 \|  \| 0.011 \|  \| 0.018 \|  \| \|  \| Col \|  \| -0.024 \| *** \|  \| -0.029 \| ** \| -0.019 \| ** \| -0.015 \|  \| \|  \|  \|  \|  \| 0.004 \|  \|  \| 0.011 \|  \| 0.006 \|  \| 0.009 \|  \| \|  \|  \|  \|  \|  \|  \|  \|  \|  \|  \|  \|  \|  \| \|  \|  \|  \|  \|  \|  \|  \|  \|  \|  \|  \|  \|  \| \| *Note*: Table figures represent probabilities. Table figures under "Marginal Effects" are marginal effects of father's education on each health measure estimated with weighted multilevel models. Table figures under "Conditional Effects" are conditional average treatment effect of respondents' education controlling for father's education. For general health, the number of unique father-child pairs = 3,186 and the total number of observations = 13,043. For psychological distress, the number of unique father-child pairs = 3,571 and the total number of observations = 20,527. < HS: less than high school; Col: college completion. ***=p<0.001 ; **=p<0.01 ; *=p<0.05; two-tailed. \| \| \| \| \| \| \| \| \| \| \| \| \| \|  \|  \| **Table 11S. Marginal and Controlled Direct Effects of Father's Education on Health: White Sample.** \| \| \| \| \| \| \| \| \| \| \| \| \|  \| \| --- \| --- \| --- \| --- \| --- \| --- \| --- \| --- \| --- \| --- \| --- \| --- \| --- \| --- \| \|  \|  \|  \|  \|  \|  \|  \|  \|  \|  \|  \|  \|  \|  \| \|  \|  \|  \|  \|  \|  \|  \|  \|  \|  \|  \|  \|  \|  \| \| **Health Outcome** \|  \| **Educ Level** \|  \| **Marginal Effect** \| \|  \| **Conditional Effect** \| \| \| \| \| \|  \| \|  \|  \|  \| **< HS** \| \| **HS** \| \| **Col** \| \|  \| \|  \|  \|  \|  \|  \|  \|  \|  \|  \|  \|  \|  \|  \|  \| \|  \|  \|  \|  \|  \|  \|  \|  \|  \|  \|  \|  \|  \|  \| \| **Poor/Fair Health** \|  \| < HS \|  \| 0.086 \| *** \|  \| 0.086 \| *** \| 0.060 \| *** \| 0.062 \| *** \|  \| \|  \|  \|  \| 0.010 \|  \|  \| 0.021 \|  \| 0.010 \|  \| 0.018 \|  \|  \| \|  \| Col \|  \| -0.023 \| * \|  \| -0.101 \| ** \| 0.021 \|  \| -0.009 \|  \|  \| \|  \|  \|  \|  \| 0.007 \|  \|  \| 0.032 \|  \| 0.011 \|  \| 0.010 \|  \|  \| \|  \|  \|  \|  \|  \|  \|  \|  \|  \|  \|  \|  \|  \|  \| \|  \|  \|  \|  \|  \|  \|  \|  \|  \|  \|  \|  \|  \|  \| \| **Severe Distress** \|  \| < HS \|  \| 0.031 \| *** \|  \| 0.067 \| *** \| 0.017 \| ** \| -0.003 \|  \|  \| \|  \|  \|  \| 0.005 \|  \|  \| 0.012 \|  \| 0.006 \|  \| 0.010 \|  \|  \| \|  \| Col \|  \| -0.014 \| ** \|  \| -0.033 \| * \| -0.007 \|  \| -0.006 \|  \|  \| \|  \|  \|  \|  \| 0.003 \|  \|  \| 0.014 \|  \| 0.006 \|  \| 0.005 \|  \|  \| \|  \|  \|  \|  \|  \|  \|  \|  \|  \|  \|  \|  \|  \|  \| \|  \|  \|  \|  \|  \|  \|  \|  \|  \|  \|  \|  \|  \|  \| \| *Note*: Table figures represent probabilities. Table figures under "Marginal Effects" are marginal effects of father's education on each health measure estimated with weighted multilevel models. Table figures under "Conditional Effects" are controlled direct effect of father's education controlling for respondents' education. For general health, the number of unique father-child pairs = 4,276 and the total number of observations = 25,267. For psychological distress, the number of unique father-child pairs = 4,807 and the total number of observations = 26,925. < HS: less than high school; Col: college completion. ***=p<0.001 ; **=p<0.01 ; *=p<0.05; two-tailed. \| \| \| \| \| \| \| \| \| \| \| \| \| \| \|  \|  \| **Table 12S. Marginal and Controlled Direct Effects of Father's Education on Health: Non-White Sample.** \| \| \| \| \| \| \| \| \| \| \| \| \| \| --- \| --- \| --- \| --- \| --- \| --- \| --- \| --- \| --- \| --- \| --- \| --- \| --- \| \|  \|  \|  \|  \|  \|  \|  \|  \|  \|  \|  \|  \|  \| \|  \|  \|  \|  \|  \|  \|  \|  \|  \|  \|  \|  \|  \| \| **Health Outcome** \|  \| **Educ Level** \|  \| **Marginal Effect** \| \|  \| **Conditional Effect** \| \| \| \| \| \| \|  \|  \|  \| **< HS** \| \| **HS** \| \| **Col** \| \| \|  \|  \|  \|  \|  \|  \|  \|  \|  \|  \|  \|  \|  \| \|  \|  \|  \|  \|  \|  \|  \|  \|  \|  \|  \|  \|  \| \| **Poor/Fair Health** \|  \| < HS \|  \| 0.044 \| *** \|  \| 0.058 \| * \| 0.044 \| ** \| 0.016 \|  \| \|  \|  \|  \| 0.014 \|  \|  \| 0.028 \|  \| 0.015 \|  \| 0.026 \|  \| \|  \| Col \|  \| -0.026 \|  \|  \| -0.003 \|  \| -0.016 \|  \| -0.030 \|  \| \|  \|  \|  \|  \| 0.018 \|  \|  \| 0.047 \|  \| 0.021 \|  \| 0.025 \|  \| \|  \|  \|  \|  \|  \|  \|  \|  \|  \|  \|  \|  \|  \| \|  \|  \|  \|  \|  \|  \|  \|  \|  \|  \|  \|  \|  \| \| **Severe Distress** \|  \| < HS \|  \| 0.030 \| *** \|  \| 0.041 \| * \| 0.020 \| * \| 0.041 \| ** \| \|  \|  \|  \| 0.007 \|  \|  \| 0.016 \|  \| 0.009 \|  \| 0.014 \|  \| \|  \| Col \|  \| -0.009 \|  \|  \| 0.003 \|  \| -0.010 \|  \| -0.009 \|  \| \|  \|  \|  \|  \| 0.008 \|  \|  \| 0.025 \|  \| 0.011 \|  \| 0.014 \|  \| \|  \|  \|  \|  \|  \|  \|  \|  \|  \|  \|  \|  \|  \| \|  \|  \|  \|  \|  \|  \|  \|  \|  \|  \|  \|  \|  \| \| *Note*: Table figures represent probabilities. Table figures under "Marginal Effects" are marginal effects of father's education on each health measure estimated with weighted multilevel models. Table figures under "Conditional Effects" are controlled direct effect of father's education controlling for respondents' education. For general health, the number of unique father-child pairs = 2,129 and the total number of observations = 11,280. For psychological distress, the number of unique father-child pairs = 2,349 and the total number of observations = 11,630. < HS: less than high school; Col: college completion. ***=p<0.001 ; **=p<0.01 ; *=p<0.05; two-tailed. \| \| \| \| \| \| \| \| \| \| \| \| \| \|  \|  \| **Table 13S. Marginal and Conditional Average Treatment Effects of Respondent's Education on Health: White Sample.** \| \| \| \| \| \| \| \| \| \| \| \| \|  \| \| --- \| --- \| --- \| --- \| --- \| --- \| --- \| --- \| --- \| --- \| --- \| --- \| --- \| --- \| \|  \|  \|  \|  \|  \|  \|  \|  \|  \|  \|  \|  \|  \|  \| \|  \|  \|  \|  \|  \|  \|  \|  \|  \|  \|  \|  \|  \|  \| \| **Health Outcome** \|  \| **Educ Level** \|  \| **Marginal Effect** \| \|  \| **Conditional Effect** \| \| \| \| \| \|  \| \|  \|  \|  \| **< HS** \| \| **HS** \| \| **Col** \| \|  \| \|  \|  \|  \|  \|  \|  \|  \|  \|  \|  \|  \|  \|  \|  \| \|  \|  \|  \|  \|  \|  \|  \|  \|  \|  \|  \|  \|  \|  \| \| **Poor/Fair Health** \|  \| < HS \|  \| 0.094 \| *** \|  \| 0.112 \| *** \| 0.083 \| *** \| -0.034 \|  \|  \| \|  \|  \|  \| 0.011 \|  \|  \| 0.016 \|  \| 0.016 \|  \| 0.030 \|  \|  \| \|  \| Col \|  \| -0.057 \| *** \|  \| -0.039 \| * \| -0.042 \| *** \| -0.067 \| *** \|  \| \|  \|  \|  \|  \| 0.006 \|  \|  \| 0.018 \|  \| 0.009 \|  \| 0.012 \|  \|  \| \|  \|  \|  \|  \|  \|  \|  \|  \|  \|  \|  \|  \|  \|  \| \|  \|  \|  \|  \|  \|  \|  \|  \|  \|  \|  \|  \|  \|  \| \| **Severe Distress** \|  \| < HS \|  \| 0.040 \| *** \|  \| 0.077 \| *** \| 0.025 \| *** \| -0.003 \|  \|  \| \|  \|  \|  \| 0.006 \|  \|  \| 0.011 \|  \| 0.008 \|  \| 0.013 \|  \|  \| \|  \| Col \|  \| -0.022 \| *** \|  \| -0.038 \| *** \| -0.018 \| *** \| -0.017 \| *** \|  \| \|  \|  \|  \|  \| 0.003 \|  \|  \| 0.011 \|  \| 0.004 \|  \| 0.006 \|  \|  \| \|  \|  \|  \|  \|  \|  \|  \|  \|  \|  \|  \|  \|  \|  \| \|  \|  \|  \|  \|  \|  \|  \|  \|  \|  \|  \|  \|  \|  \| \| *Note*: Table figures represent probabilities. Table figures under "Marginal Effects" are marginal effects of father's education on each health measure estimated with weighted multilevel models. Table figures under "Conditional Effects" are conditional average treatment effect of respondents' education controlling for father's education. For general health, the number of unique father-child pairs = 4,276 and the total number of observations = 25,267. For psychological distress, the number of unique father-child pairs = 4,807 and the total number of observations = 26,925. < HS: less than high school; Col: college completion. ***=p<0.001 ; **=p<0.01 ; *=p<0.05; two-tailed. \| \| \| \| \| \| \| \| \| \| \| \| \| \| \|  \|  \| **Table 14S. Marginal and Conditional Average Treatment Effects of Respondent's Education on Health: Non-White Sample.** \| \| \| \| \| \| \| \| \| \| \| \| \| \| --- \| --- \| --- \| --- \| --- \| --- \| --- \| --- \| --- \| --- \| --- \| --- \| --- \| \|  \|  \|  \|  \|  \|  \|  \|  \|  \|  \|  \|  \|  \| \|  \|  \|  \|  \|  \|  \|  \|  \|  \|  \|  \|  \|  \| \| **Health Outcome** \|  \| **Educ Level** \|  \| **Marginal Effect** \| \|  \| **Conditional Effect** \| \| \| \| \| \| \|  \|  \|  \| **< HS** \| \| **HS** \| \| **Col** \| \| \|  \|  \|  \|  \|  \|  \|  \|  \|  \|  \|  \|  \|  \| \|  \|  \|  \|  \|  \|  \|  \|  \|  \|  \|  \|  \|  \| \| **Poor/Fair Health** \|  \| < HS \|  \| 0.087 \| *** \|  \| 0.090 \| *** \| 0.068 \| ** \| 0.089 \|  \| \|  \|  \|  \| 0.015 \|  \|  \| 0.021 \|  \| 0.022 \|  \| 0.051 \|  \| \|  \| Col \|  \| -0.058 \| *** \|  \| -0.067 \| ** \| -0.043 \| ** \| -0.060 \|  \| \|  \|  \|  \|  \| 0.012 \|  \|  \| 0.025 \|  \| 0.016 \|  \| 0.032 \|  \| \|  \|  \|  \|  \|  \|  \|  \|  \|  \|  \|  \|  \|  \| \|  \|  \|  \|  \|  \|  \|  \|  \|  \|  \|  \|  \|  \| \| **Severe Distress** \|  \| < HS \|  \| 0.044 \| *** \|  \| 0.052 \| *** \| 0.030 \| ** \| 0.052 \|  \| \|  \|  \|  \| 0.008 \|  \|  \| 0.013 \|  \| 0.011 \|  \| 0.027 \|  \| \|  \| Col \|  \| -0.014 \| * \|  \| 0.004 \|  \| -0.015 \|  \| -0.014 \|  \| \|  \|  \|  \|  \| 0.006 \|  \|  \| 0.013 \|  \| 0.008 \|  \| 0.017 \|  \| \|  \|  \|  \|  \|  \|  \|  \|  \|  \|  \|  \|  \|  \| \|  \|  \|  \|  \|  \|  \|  \|  \|  \|  \|  \|  \|  \| \| *Note*: Table figures represent probabilities. Table figures under "Marginal Effects" are marginal effects of father's education on each health measure estimated with weighted multilevel models. Table figures under "Conditional Effects" are conditional average treatment effect of respondents' education controlling for father's education. For general health, the number of unique father-child pairs = 2,129 and the total number of observations = 11,280. For psychological distress, the number of unique father-child pairs = 2,349 and the total number of observations = 11,630. < HS: less than high school; Col: college completion. ***=p<0.001; **=p<0.01; *=p<0.05; two-tailed. \| \| \| \| \| \| \| \| \| \| \| \| \|  \|  \| **Table 15S. Marginal and Controlled Direct Effects of Father's Education on Health, the Panel Study of Income Dynamics: the 1950s Cohort.** \| \| \| \| \| \| \| \| \| \| \| \| \| \| --- \| --- \| --- \| --- \| --- \| --- \| --- \| --- \| --- \| --- \| --- \| --- \| --- \| \|  \|  \|  \|  \|  \|  \|  \|  \|  \|  \|  \|  \|  \| \|  \|  \|  \|  \|  \|  \|  \|  \|  \|  \|  \|  \|  \| \| **Health Outcome** \|  \| **Educ Level** \|  \| **Marginal Effect** \| \|  \| **Conditional Effect** \| \| \| \| \| \| \|  \|  \|  \| **< HS** \| \| **HS** \| \| **Col** \| \| \|  \|  \|  \|  \|  \|  \|  \|  \|  \|  \|  \|  \|  \| \|  \|  \|  \|  \|  \|  \|  \|  \|  \|  \|  \|  \|  \| \| **Poor/Fair Health** \|  \| < HS \|  \| 0.110 \| *** \|  \| 0.065 \|  \| 0.092 \| *** \| -0.081 \| * \| \|  \|  \|  \| 0.018 \|  \|  \| 0.067 \|  \| 0.020 \|  \| 0.034 \|  \| \|  \| Col \|  \| 0.005 \|  \|  \| -0.240 \|  \| 0.048 \|  \| 0.007 \|  \| \|  \|  \|  \|  \| 0.022 \|  \|  \| 0.192 \|  \| 0.032 \|  \| 0.027 \|  \| \|  \|  \|  \|  \|  \|  \|  \|  \|  \|  \|  \|  \|  \| \|  \|  \|  \|  \|  \|  \|  \|  \|  \|  \|  \|  \|  \| \| **Severe Distress** \|  \| < HS \|  \| 0.043 \| *** \|  \| 0.083 \| * \| 0.034 \| ** \| 0.029 \|  \| \|  \|  \|  \| 0.010 \|  \|  \| 0.041 \|  \| 0.013 \|  \| 0.020 \|  \| \|  \| Col \|  \| 0.011 \|  \|  \| -0.092 \|  \| 0.016 \|  \| 0.017 \|  \| \|  \|  \|  \|  \| 0.012 \|  \|  \| 0.116 \|  \| 0.020 \|  \| 0.016 \|  \| \|  \|  \|  \|  \|  \|  \|  \|  \|  \|  \|  \|  \|  \| \|  \|  \|  \|  \|  \|  \|  \|  \|  \|  \|  \|  \|  \| \| *Note*: Table figures represent probabilities. Table figures under "Marginal Effects" are marginal effects of father's education on each health measure estimated with weighted multilevel models. Table figures under "Conditional Effects" are controlled direct effect of father's education controlling for respondents' education. For general health, the number of unique father-child pairs = 892 and the total number of observations = 11,000. For psychological distress, the number of unique father-child pairs = 934 and the total number of observations = 6,308. High school graduate is omitted as the reference group for all outcomes. < HS: less than high school; Col: college completion. ***=p<0.001; **=p<0.01; *=p<0.05; two-tailed. \| \| \| \| \| \| \| \| \| \| \| \| \| \|  \|  \| **Table 16S. Marginal and Conditional Average Treatment Effects of Respondent's Education on Health, Conditional on Father's Education, the Panel Study of Income Dynamics: the 1950s Cohort.** \| \| \| \| \| \| \| \| \| \| \| \| \| \| --- \| --- \| --- \| --- \| --- \| --- \| --- \| --- \| --- \| --- \| --- \| --- \| --- \| \|  \|  \|  \|  \|  \|  \|  \|  \|  \|  \|  \|  \|  \| \|  \|  \|  \|  \|  \|  \|  \|  \|  \|  \|  \|  \|  \| \| **Health Outcome** \|  \| **Educ Level** \|  \| **Marginal Effect** \| \|  \| **Conditional Effect** \| \| \| \| \| \| \|  \|  \|  \| **< HS** \| \| **HS** \| \| **Col** \| \| \|  \|  \|  \|  \|  \|  \|  \|  \|  \|  \|  \|  \|  \| \|  \|  \|  \|  \|  \|  \|  \|  \|  \|  \|  \|  \|  \| \| **Poor/Fair Health** \|  \| < HS \|  \| 0.192 \| *** \|  \| 0.173 \| *** \| 0.202 \| *** \| -0.088 \|  \| \|  \|  \|  \| 0.030 \|  \|  \| 0.036 \|  \| 0.059 \|  \| 0.187 \|  \| \|  \| Col \|  \| -0.071 \| *** \|  \| -0.047 \|  \| -0.039 \|  \| -0.082 \| * \| \|  \|  \|  \|  \| 0.015 \|  \|  \| 0.031 \|  \| 0.023 \|  \| 0.034 \|  \| \|  \|  \|  \|  \|  \|  \|  \|  \|  \|  \|  \|  \|  \| \|  \|  \|  \|  \|  \|  \|  \|  \|  \|  \|  \|  \|  \| \| **Severe Distress** \|  \| < HS \|  \| 0.115 \| *** \|  \| 0.125 \| *** \| 0.075 \| * \| -0.033 \|  \| \|  \|  \|  \| 0.018 \|  \|  \| 0.021 \|  \| 0.034 \|  \| 0.111 \|  \| \|  \| Col \|  \| -0.015 \|  \|  \| -0.013 \|  \| -0.009 \|  \| -0.010 \|  \| \|  \|  \|  \|  \| 0.009 \|  \|  \| 0.017 \|  \| 0.014 \|  \| 0.021 \|  \| \|  \|  \|  \|  \|  \|  \|  \|  \|  \|  \|  \|  \|  \| \|  \|  \|  \|  \|  \|  \|  \|  \|  \|  \|  \|  \|  \| \| *Note*: Table figures represent probabilities. Table figures under "Marginal Effects" are marginal effects of father's education on each health measure estimated with weighted multilevel models. Table figures under "Conditional Effects" are conditional average treatment effect of respondents' education controlling for father's education. For general health, the number of unique father-child pairs = 892 and the total number of observations = 11,000. For psychological distress, the number of unique father-child pairs = 934 and the total number of observations = 6,308. High school graduate is omitted as the reference group for all outcomes. < HS: less than high school; Col: college completion. ***=p<0.001; **=p<0.01; *=p<0.05; two-tailed. \| \| \| \| \| \| \| \| \| \| \| \| \| \|  \|  \| **Table 17S. Marginal and Controlled Direct Effects of Father's Education on Health, the Panel Study of Income Dynamics: the 1960s Cohort.** \| \| \| \| \| \| \| \| \| \| \| \| \| \| --- \| --- \| --- \| --- \| --- \| --- \| --- \| --- \| --- \| --- \| --- \| --- \| --- \| \|  \|  \|  \|  \|  \|  \|  \|  \|  \|  \|  \|  \|  \| \|  \|  \|  \|  \|  \|  \|  \|  \|  \|  \|  \|  \|  \| \| **Health Outcome** \|  \| **Educ Level** \|  \| **Marginal Effect** \| \|  \| **Conditional Effect** \| \| \| \| \| \| \|  \|  \|  \| **< HS** \| \| **HS** \| \| **Col** \| \| \|  \|  \|  \|  \|  \|  \|  \|  \|  \|  \|  \|  \|  \| \|  \|  \|  \|  \|  \|  \|  \|  \|  \|  \|  \|  \|  \| \| **Poor/Fair Health** \|  \| < HS \|  \| 0.081 \| *** \|  \| 0.021 \|  \| 0.074 \| *** \| 0.022 \|  \| \|  \|  \|  \| 0.014 \|  \|  \| 0.049 \|  \| 0.016 \|  \| 0.029 \|  \| \|  \| Col \|  \| -0.003 \|  \|  \| 0.032 \|  \| 0.038 \|  \| -0.012 \|  \| \|  \|  \|  \|  \| 0.016 \|  \|  \| 0.151 \|  \| 0.022 \|  \| 0.020 \|  \| \|  \|  \|  \|  \|  \|  \|  \|  \|  \|  \|  \|  \|  \| \|  \|  \|  \|  \|  \|  \|  \|  \|  \|  \|  \|  \|  \| \| **Severe Distress** \|  \| < HS \|  \| 0.016 \| * \|  \| 0.052 \| * \| 0.007 \|  \| -0.008 \|  \| \|  \|  \|  \| 0.007 \|  \|  \| 0.026 \|  \| 0.009 \|  \| 0.015 \|  \| \|  \| Col \|  \| -0.013 \|  \|  \| -0.039 \|  \| -0.011 \|  \| -0.010 \|  \| \|  \|  \|  \|  \| 0.008 \|  \|  \| 0.074 \|  \| 0.012 \|  \| 0.011 \|  \| \|  \|  \|  \|  \|  \|  \|  \|  \|  \|  \|  \|  \|  \| \|  \|  \|  \|  \|  \|  \|  \|  \|  \|  \|  \|  \|  \| \| *Note*: Table figures represent probabilities. Table figures under "Marginal Effects" are marginal effects of father's education on each health measure estimated with weighted multilevel models. Table figures under "Conditional Effects" are controlled direct effect of father's education controlling for respondents' education. For general health, the number of unique father-child pairs = 966 and the total number of observations = 10,432. For psychological distress, the number of unique father-child pairs = 1,023 and the total number of observations = 6,903. High school graduate is omitted as the reference group for all outcomes. < HS: less than high school; Col: college completion. ***=p<0.001; **=p<0.01; *=p<0.05; two-tailed. \| \| \| \| \| \| \| \| \| \| \| \| \| \|  \|  \| **Table 18S. Marginal and Conditional Average Treatment Effects of Respondent's Education on Health, Conditional on Father's Education, the Panel Study of Income Dynamics: the 1960s Cohort.** \| \| \| \| \| \| \| \| \| \| \| \| \| \| --- \| --- \| --- \| --- \| --- \| --- \| --- \| --- \| --- \| --- \| --- \| --- \| --- \| \|  \|  \|  \|  \|  \|  \|  \|  \|  \|  \|  \|  \|  \| \|  \|  \|  \|  \|  \|  \|  \|  \|  \|  \|  \|  \|  \| \| **Health Outcome** \|  \| **Educ Level** \|  \| **Marginal Effect** \| \|  \| **Conditional Effect** \| \| \| \| \| \| \|  \|  \|  \| **< HS** \| \| **HS** \| \| **Col** \| \| \|  \|  \|  \|  \|  \|  \|  \|  \|  \|  \|  \|  \|  \| \|  \|  \|  \|  \|  \|  \|  \|  \|  \|  \|  \|  \|  \| \| **Poor/Fair Health** \|  \| < HS \|  \| 0.079 \| *** \|  \| 0.045 \|  \| 0.090 \|  \| 0.093 \|  \| \|  \|  \|  \| 0.022 \|  \|  \| 0.027 \|  \| 0.042 \|  \| 0.148 \|  \| \|  \| Col \|  \| -0.059 \| *** \|  \| -0.076 \| ** \| -0.023 \|  \| -0.076 \| ** \| \|  \|  \|  \|  \| 0.012 \|  \|  \| 0.028 \|  \| 0.016 \|  \| 0.025 \|  \| \|  \|  \|  \|  \|  \|  \|  \|  \|  \|  \|  \|  \|  \| \|  \|  \|  \|  \|  \|  \|  \|  \|  \|  \|  \|  \|  \| \| **Severe Distress** \|  \| < HS \|  \| 0.044 \| *** \|  \| 0.053 \| *** \| 0.013 \|  \| -0.017 \|  \| \|  \|  \|  \| 0.012 \|  \|  \| 0.015 \|  \| 0.022 \|  \| 0.076 \|  \| \|  \| Col \|  \| -0.014 \| * \|  \| -0.025 \|  \| -0.008 \|  \| -0.006 \|  \| \|  \|  \|  \|  \| 0.006 \|  \|  \| 0.015 \|  \| 0.009 \|  \| 0.014 \|  \| \|  \|  \|  \|  \|  \|  \|  \|  \|  \|  \|  \|  \|  \| \|  \|  \|  \|  \|  \|  \|  \|  \|  \|  \|  \|  \|  \| \| Note: Table figures represent probabilities. Table figures under "Marginal Effects" are average effects of father's education on each health measure estimated with weighted multilevel models. Table figures under "Conditional Effects" are conditional average treatment effect of respondents' education controlling for father's education. For general health, the number of unique father-child pairs = 6,405 and the total number of observations = 37,398. For psychological distress, the number of unique father-child pairs = 7,156 and the total number of observations = 39,725. High school completion is omitted as the reference group for all analyses. < HS: less than high school; Col: college completion. ***=p<0.001; **=p<0.01; *=p<0.05; two-tailed. \| \| \| \| \| \| \| \| \| \| \| \| \| \|  \|  \| **Table 19S. Marginal and Controlled Direct Effects of Mother's Education on Health, the Panel Study of Income Dynamics, 1968-2017.** \| \| \| \| \| \| \| \| \| \| \| \| \| \| --- \| --- \| --- \| --- \| --- \| --- \| --- \| --- \| --- \| --- \| --- \| --- \| --- \| \|  \|  \|  \|  \|  \|  \|  \|  \|  \|  \|  \|  \|  \| \|  \|  \|  \|  \|  \|  \|  \|  \|  \|  \|  \|  \|  \| \| **Health Outcome** \|  \| **Educ Level** \|  \| **Marginal Effect** \| \|  \| **Conditional Effect** \| \| \| \| \| \| \|  \|  \|  \| **< HS** \| \| **HS** \| \| **Col** \| \| \|  \|  \|  \|  \|  \|  \|  \|  \|  \|  \|  \|  \|  \| \|  \|  \|  \|  \|  \|  \|  \|  \|  \|  \|  \|  \|  \| \| **Poor/Fair Health** \|  \| < HS \|  \| 0.072 \| *** \|  \| 0.058 \| * \| 0.058 \| *** \| 0.027 \|  \| \|  \|  \|  \| 0.009 \|  \|  \| 0.024 \|  \| 0.010 \|  \| 0.020 \|  \| \|  \| Col \|  \| -0.038 \| *** \|  \| -0.094 \| ** \| -0.007 \|  \| -0.019 \|  \| \|  \|  \|  \|  \| 0.008 \|  \|  \| 0.033 \|  \| 0.012 \|  \| 0.010 \|  \| \|  \|  \|  \|  \|  \|  \|  \|  \|  \|  \|  \|  \|  \| \|  \|  \|  \|  \|  \|  \|  \|  \|  \|  \|  \|  \|  \| \| **Severe Distress** \|  \| < HS \|  \| 0.027 \| *** \|  \| 0.034 \| *** \| 0.019 \| *** \| 0.011 \|  \| \|  \|  \|  \| 0.004 \|  \|  \| 0.011 \|  \| 0.005 \|  \| 0.009 \|  \| \|  \| Col \|  \| -0.016 \| *** \|  \| -0.057 \| *** \| -0.007 \|  \| -0.007 \|  \| \|  \|  \|  \|  \| 0.003 \|  \|  \| 0.013 \|  \| 0.006 \|  \| 0.005 \|  \| \|  \|  \|  \|  \|  \|  \|  \|  \|  \|  \|  \|  \|  \| \|  \|  \|  \|  \|  \|  \|  \|  \|  \|  \|  \|  \|  \| \| *Note*: Table figures represent probabilities. Table figures under "Marginal Effects" are average effects of mother's education on each health measure estimated with weighted multilevel models. Table figures under "Conditional Effects" are controlled direct effect of mother's education controlling for respondents' education. For general health, the number of unique mother-child pairs = 6,405 and the total number of observations = 37,398. For psychological distress, the number of unique mother-child pairs = 7,156 and the total number of observations = 39,725. High school completion is omitted as the reference group for all analyses. < HS: less than high school; Col: college completion. ***=p<0.001; **=p<0.01; *=p<0.05; two-tailed. \| \| \| \| \| \| \| \| \| \| \| \| \| \|  \|  \| **Table 20S. Marginal and Conditional (on Mother's Education) Average Treatment Effects of Own Education on Health, the Panel Study of Income Dynamics, 1968-2017.** \| \| \| \| \| \| \| \| \| \| \| \| \| \| --- \| --- \| --- \| --- \| --- \| --- \| --- \| --- \| --- \| --- \| --- \| --- \| --- \| \|  \|  \|  \|  \|  \|  \|  \|  \|  \|  \|  \|  \|  \| \|  \|  \|  \|  \|  \|  \|  \|  \|  \|  \|  \|  \|  \| \| **Health Outcome** \|  \| **Educ Level** \|  \| **Marginal Effect** \| \|  \| **Conditional Effect** \| \| \| \| \| \| \|  \|  \|  \| **< HS** \| \| **HS** \| \| **Col** \| \| \|  \|  \|  \|  \|  \|  \|  \|  \|  \|  \|  \|  \|  \| \|  \|  \|  \|  \|  \|  \|  \|  \|  \|  \|  \|  \|  \| \| **Poor/Fair Health** \|  \| < HS \|  \| 0.081 \| *** \|  \| 0.078 \| *** \| 0.078 \| *** \| -0.009 \|  \| \|  \|  \|  \| 0.010 \|  \|  \| 0.021 \|  \| 0.015 \|  \| 0.033 \|  \| \|  \| Col \|  \| -0.068 \| *** \|  \| -0.083 \| *** \| -0.053 \| *** \| -0.064 \| *** \| \|  \|  \|  \|  \| 0.006 \|  \|  \| 0.021 \|  \| 0.008 \|  \| 0.014 \|  \| \|  \|  \|  \|  \|  \|  \|  \|  \|  \|  \|  \|  \|  \| \|  \|  \|  \|  \|  \|  \|  \|  \|  \|  \|  \|  \|  \| \| **Severe Distress** \|  \| < HS \|  \| 0.042 \| *** \|  \| 0.056 \| *** \| 0.039 \| *** \| 0.006 \|  \| \|  \|  \|  \| 0.005 \|  \|  \| 0.009 \|  \| 0.007 \|  \| 0.012 \|  \| \|  \| Col \|  \| -0.022 \| *** \|  \| -0.023 \| * \| -0.017 \| *** \| -0.018 \| ** \| \|  \|  \|  \|  \| 0.003 \|  \|  \| 0.009 \|  \| 0.004 \|  \| 0.006 \|  \| \|  \|  \|  \|  \|  \|  \|  \|  \|  \|  \|  \|  \|  \| \|  \|  \|  \|  \|  \|  \|  \|  \|  \|  \|  \|  \|  \| \| *Note*: Table figures represent probabilities. Table figures under "Marginal Effects" are average effects of education on each health measure estimated with weighted multilevel model. Table figures under "Conditional Effects" are conditional average treatment effects of respondents' education controlling for mother's education. For general health, the number of unique mother-child pairs = 6,405 and the total number of observations = 37,398. For psychological distress, the number of unique mother-child pairs = 7,156 and the total number of observations = 39,725. High school completion is omitted as the reference group for all analyses. < HS: less than high school; Col: college completion. ***=p<0.001; **=p<0.01; *=p<0.05; two-tailed. \| \| \| \| \| \| \| \| \| \| \| \| \| \|  \|   **TECHNICAL APPENDIX**  **Weighting Diagnostics and Sensitivity Analysis**  The estimated education effects on the health measures are unbiased only when weighting balances measured covariates between education groups. We used weighted standardized differences to assess if the weights have achieved sufficient covariate balance and reported the diagnostics in Table 3A. For both parent- and the respondent-level covariates, all standardized differences have numeric values of .04 or less, which is less than the conventional threshold of .1 and thus indicates a good balance in covariates among education groups.  We also conducted sensitivity analysis to examine the potential influence of unmeasured confounding on the observed education-health relationship. We used the E-value developed by Ding and VanderWeele (2016) to estimate a minimum strength of unmeasured confounding that could explain away the observed education effects on health after conditioning on all observed confounders. The advantage of the E-value approach is that it does not assume the strength, form, or pattern of the association of the unobserved confounder(s). As depicted in Appendix Figures 1A and 2A, the estimated E-values are 2.3 to 2.43 for own education effects and 1.78 to 2.25 for the parent’s education effects on the two outcomes.  Consider own education effect on self-reported health, where the E-value is 2.3. If an unobserved confounder can entirely account for the observed causal effect of own education on self-reported health, conditioning on all other observed confounders, the unobserved confounder would need to more than double the probability of completing college (increase by 130%) and more than double the probability of reporting poor or fair health status (increase by 130%). In comparison, a common confounder, like city residence, is only associated with 6% and 1% increase in the probability of receiving treatment (e.g., college completion) and attaining outcome (e.g., poor or fair health) respectively after other confounders are accounted for. That is, for own education effect on self-reported health, an unobserved confounder must be about 22 (130/6) to 130 (130/1) times stronger than city residence to nullify the result. The evidence for causality from the E-value is thus reasonably strong—although more moderate for parent’s education on self-reported health with values of 1.78 and 2.25—as it would take substantially strong unmeasured confounding to reduce the observed education effect to null. See more details about the E-value in Ding and VanderWeele (2016) and VanderWeele and Ding (2017).  Lastly, we note that the sensitivity analysis tests whether the estimated effects, including CDEs of parental education and the CATEs of own education, are sensitive to the existence of unmeasured confounder. Because the CDEs and CATEs are estimated in separate models, sensitivity analyses need to be conducted separately, that is, for each sensitivity analysis, we can only test the sensitivity of one effect.  **Additional Details about the Estimands**  The controlled direct effect (CDE) is non-parametrically identified as (Robins and Greenland 1992):  $E[Y(1,r)-Y(0,r)]=\sum_{X_{P}} \sum_{X_{R}} E[Y\vert x_{P},x_{R},P=1,r]P(x_{R}\vert P=1,x_{P})P(x_{P})$ $-\sum_{X_{P}} \sum_{X_{R}} E[Y\vert x_{P},x_{R},P=0,r]P(x_{R}\vert P=0,x_{P})P(x_{P})$. (1)  The identification of Equation (1) differs from the standard regression model in that they marginalize out the respondent’s pre-education confounders $X_{R}$’s in distinct ways. The identification of Equation (1) acknowledges the effects of parent-level covariates $X_{P}$ and parent’s education $P$ on respondent’s childhood health and socioeconomic factors $X_{R}$. In contrast, the standard regression model weighs the two conditional expectations using the same distribution of $X_{R}$, which is inconsistent with empirical evidence that the respondents with different parental education levels have different distributions in childhood health and socioeconomic factors $X_{R}$ (see Smith 2003 for detailed discussions).  Additionally, under the potential outcome framework of causal analysis, there are two approaches to mediation analysis for observational data. One is natural direct and indirect effect (NDE and NIE respectively). The other is CDE (Pearl 2022; Robins and Greenland 1992). The NIE and the NDE are generally unidentifiable in the context of intergenerational education due to respondent-level covariates $X_{R}$’s—a set of intermediate confounders (Avin, Shpitser, and Pearl 2005), so we focus on the CDEs in this study.  **Implementation and Example R Code**  Here we provide a step-by-step description about how to implement the weighted multilevel marginal structural model (MMSM), along with example R code. The procedure consists of two steps. The first step is to compute separate weights for parent’s and own education based on covariate balancing propensity score (CBPS). The second step is to estimate the MMSM with weights computed in Step 1.  Step 1. To compute covariate balancing propensity score (CBPS) weights for parental education, first treat parental education as the outcome and pre-education factors such as the parent’s childhood health socioeconomic conditions as predictors in a multinomial logistic regression model. Use this set of results to predict the parent’s probability of achieving his or her observed educational level while balancing covariate distributions for the same level of propensity score. The CBPS weights for parental education are the inverse of these probabilities. The CBPS weights for own education can be computed in a similar fashion (i.e., use the respondent’s childhood socioeconomic and health conditions as predictors to predict their educational level while balancing covariate distributions). The CBPS weights used in this study were computed using the R package CBPS.  Example R code for implementing Step 1:  *#Estimate CBPS for parental and own education*  *#Note the control variables are different for parents and respondents.*  *library(CBPS)*  *ps_parent <- CBPS(edu_p ~ controls_parent, data)*  *ps_own <- CBPS(edu ~ control_own + controls_parent, data)*  *#Store weights in a data frame for estimating parent’s and own education effects*  *data$parent_weight <- ps_parent[[“weights”]]*ps_own[[“weights”]]*  *data$own_weight <- ps_own[[“weights”]]*  Step 2. Estimate the multilevel marginal structural models (MMSM) using the CBPS weights from Step 1. Different sets of weights are used to estimate the effect of parental education and own education on each outcome (i.e., self-reported health and severe psychological distress). See Equations (1)-(8) in the main text for model specifications. The MMSMs in the present study were estimated using the R package lme4.  Example R code for implementing Step 2:  *library(lme4)*  *#Estimating ATE of own education on health*  *lmer(health~ 1 + edu + cohort + (1\|pid), data, weights = own_weight)*  *#Estimating ATE of parental education on health*  *lmer(health~ 1 + edu_p + cohort + (1\|pid), data, weights = ps_parent[[“weights”]])*  *#Estimating CATE of own education by parental education*  *lmer(health ~ 1 + edu*edu_p + cohort + (1\|pid), data, weights = own_weight)*  *#Estimating CDE of parental education by own education*  *lmer(health ~ 1 + edu*edu_p + cohort + (1\|pid), data, weights = parent_weight)*  *#Estimating own education effect over the life course*  *lmer(health ~ 1 + age*edu + cohort + (1\|pid), data, weights = own_weight)*  *#Estimating parental education effect over the life course*  *lmer(health ~ 1 + age*edu_p + cohort + (1\|pid), data, weights = ps_parent[[“weights”]])*  **References**  Avin, Chen, Ilya Shpitser, and Judea Pearl. 2005. “Identifiability of Path-Specific Effects.” https://escholarship.org/uc/item/45x689gq (April 11, 2022).  Ding, Peng, and Tyler J. VanderWeele. 2016. “Sensitivity Analysis Without Assumptions.” *Epidemiology (Cambridge, Mass.)* 27(3): 368–77.  Pearl, Judea. 2022. “Direct and Indirect Effects.” In *Probabilistic and Causal Inference: The Works of Judea Pearl*, eds. Hector Geffner, Rina Dechter, and Joseph Halpern. New York, NY, USA: Association for Computing Machinery, 373–92.  Robins, J. M., and S. Greenland. 1992. “Identifiability and Exchangeability for Direct and Indirect Effects.” *Epidemiology (Cambridge, Mass.)* 3(2): 143–55.  Smith, Herbert L. 2003. “Some Thoughts on Causation as It Relates to Demography and Population Studies.” *Population and Development Review* 29(3): 459–69.  VanderWeele, Tyler J., and Peng Ding. 2017. “Sensitivity Analysis in Observational Research: Introducing the E-Value.” *Annals of Internal Medicine* 167(4): 268–74. |
| --- | --- | --- | --- | --- | --- | --- | --- | --- | --- | --- | --- | --- | --- | --- | --- | --- | --- | --- | --- | --- | --- | --- | --- | --- | --- | --- | --- | --- | --- | --- | --- | --- | --- | --- | --- | --- | --- | --- | --- | --- | --- | --- | --- | --- | --- | --- | --- | --- | --- | --- | --- | --- | --- | --- | --- | --- | --- | --- | --- | --- | --- | --- | --- | --- | --- | --- | --- | --- | --- | --- | --- | --- | --- | --- | --- | --- | --- | --- | --- | --- | --- | --- | --- | --- | --- | --- | --- | --- | --- | --- | --- | --- | --- | --- | --- | --- | --- | --- | --- | --- | --- | --- | --- | --- | --- | --- | --- | --- | --- | --- | --- | --- | --- | --- | --- | --- | --- | --- | --- | --- | --- | --- | --- | --- | --- | --- | --- | --- | --- | --- | --- | --- | --- | --- | --- | --- | --- | --- | --- | --- | --- | --- | --- | --- | --- | --- | --- | --- | --- | --- | --- | --- | --- | --- | --- | --- | --- | --- | --- | --- | --- | --- | --- | --- | --- | --- | --- | --- | --- | --- | --- | --- | --- | --- | --- | --- | --- | --- | --- | --- | --- | --- | --- | --- | --- | --- | --- | --- | --- | --- | --- | --- | --- | --- | --- | --- | --- | --- | --- | --- | --- | --- | --- | --- | --- | --- | --- | --- | --- | --- | --- | --- | --- | --- | --- | --- | --- | --- | --- | --- | --- | --- | --- | --- | --- | --- | --- | --- | --- | --- | --- | --- | --- | --- | --- | --- | --- | --- | --- | --- | --- | --- | --- | --- | --- | --- | --- | --- | --- | --- | --- | --- | --- | --- | --- | --- | --- | --- | --- | --- | --- | --- | --- | --- | --- | --- | --- | --- | --- | --- | --- | --- | --- | --- | --- | --- | --- | --- | --- | --- | --- | --- | --- | --- | --- | --- | --- | --- | --- | --- | --- | --- | --- | --- | --- | --- | --- | --- | --- | --- | --- | --- | --- | --- | --- | --- | --- | --- | --- | --- | --- | --- | --- | --- | --- | --- | --- | --- | --- | --- | --- | --- | --- | --- | --- | --- | --- | --- | --- | --- | --- | --- | --- | --- | --- | --- | --- | --- | --- | --- | --- | --- | --- | --- | --- | --- | --- | --- | --- | --- | --- | --- | --- | --- | --- | --- | --- | --- | --- | --- | --- | --- | --- | --- | --- | --- | --- | --- | --- | --- | --- | --- | --- | --- | --- | --- | --- | --- | --- | --- | --- | --- | --- | --- | --- | --- | --- | --- | --- | --- | --- | --- | --- | --- | --- | --- | --- | --- | --- | --- | --- | --- | --- | --- | --- | --- | --- | --- | --- | --- | --- | --- | --- | --- | --- | --- | --- | --- | --- | --- | --- | --- | --- | --- | --- | --- | --- | --- | --- | --- | --- | --- | --- | --- | --- | --- | --- | --- | --- | --- | --- | --- | --- | --- | --- | --- | --- | --- | --- | --- | --- | --- | --- | --- | --- | --- | --- | --- | --- | --- | --- | --- | --- | --- | --- | --- | --- | --- | --- | --- | --- | --- | --- | --- | --- | --- | --- | --- | --- | --- | --- | --- | --- | --- | --- | --- | --- | --- | --- | --- | --- | --- | --- | --- | --- | --- | --- | --- | --- | --- | --- | --- | --- | --- | --- | --- | --- | --- | --- | --- | --- | --- | --- | --- | --- | --- | --- | --- | --- | --- | --- | --- | --- | --- | --- | --- | --- | --- | --- | --- | --- | --- | --- | --- | --- | --- | --- | --- | --- | --- | --- | --- | --- | --- | --- | --- | --- | --- | --- | --- | --- | --- | --- | --- | --- | --- | --- | --- | --- | --- | --- | --- | --- | --- | --- | --- | --- | --- | --- | --- | --- | --- | --- | --- | --- | --- | --- | --- | --- | --- | --- | --- | --- | --- | --- | --- | --- | --- | --- | --- | --- | --- | --- | --- | --- | --- | --- | --- | --- | --- | --- | --- | --- | --- | --- | --- | --- | --- | --- | --- | --- | --- | --- | --- | --- | --- | --- | --- | --- | --- | --- | --- | --- | --- | --- | --- | --- | --- | --- | --- | --- | --- | --- | --- | --- | --- | --- | --- | --- | --- | --- | --- | --- | --- | --- | --- | --- | --- | --- | --- | --- | --- | --- | --- | --- | --- | --- | --- | --- | --- | --- | --- | --- | --- | --- | --- | --- | --- | --- | --- | --- | --- | --- | --- | --- | --- | --- | --- | --- | --- | --- | --- | --- | --- | --- | --- | --- | --- | --- | --- | --- | --- | --- | --- | --- | --- | --- | --- | --- | --- | --- | --- | --- | --- | --- | --- | --- | --- | --- | --- | --- | --- | --- | --- | --- | --- | --- | --- | --- | --- | --- | --- | --- | --- | --- | --- | --- | --- | --- | --- | --- | --- | --- | --- | --- | --- | --- | --- | --- | --- | --- | --- | --- | --- | --- | --- | --- | --- | --- | --- | --- | --- | --- | --- | --- | --- | --- | --- | --- | --- | --- | --- | --- | --- | --- | --- | --- | --- | --- | --- | --- | --- | --- | --- | --- | --- | --- | --- | --- | --- | --- | --- | --- | --- | --- | --- | --- | --- | --- | --- | --- | --- | --- | --- | --- | --- | --- | --- | --- | --- | --- | --- | --- | --- | --- | --- | --- | --- | --- | --- | --- | --- | --- | --- | --- | --- | --- | --- | --- | --- | --- | --- | --- | --- | --- | --- | --- | --- | --- | --- | --- | --- | --- | --- | --- | --- | --- | --- | --- | --- | --- | --- | --- | --- | --- | --- | --- | --- | --- | --- | --- | --- | --- | --- | --- | --- | --- | --- | --- | --- | --- | --- | --- | --- | --- | --- | --- | --- | --- | --- | --- | --- | --- | --- | --- | --- | --- | --- | --- | --- | --- | --- | --- | --- | --- | --- | --- | --- | --- | --- | --- | --- | --- | --- | --- | --- | --- | --- | --- | --- | --- | --- | --- | --- | --- | --- | --- | --- | --- | --- | --- | --- | --- | --- | --- | --- | --- | --- | --- | --- | --- | --- | --- | --- | --- | --- | --- | --- | --- | --- | --- | --- | --- | --- | --- | --- | --- | --- | --- | --- | --- | --- | --- | --- | --- | --- | --- | --- | --- | --- | --- | --- | --- | --- | --- | --- | --- | --- | --- | --- | --- | --- | --- | --- | --- | --- | --- | --- | --- | --- | --- | --- | --- | --- | --- | --- | --- | --- | --- | --- | --- | --- | --- | --- | --- | --- | --- | --- | --- | --- | --- | --- | --- | --- | --- | --- | --- | --- | --- | --- | --- | --- | --- | --- | --- | --- | --- | --- | --- | --- | --- | --- | --- | --- | --- | --- | --- | --- | --- | --- | --- | --- | --- | --- | --- | --- | --- | --- | --- | --- | --- | --- | --- | --- | --- | --- | --- | --- | --- | --- | --- | --- | --- | --- | --- | --- | --- | --- | --- | --- | --- | --- | --- | --- | --- | --- | --- | --- | --- | --- | --- | --- | --- | --- | --- | --- | --- | --- | --- | --- | --- | --- | --- | --- | --- | --- | --- | --- | --- | --- | --- | --- | --- | --- | --- | --- | --- | --- | --- | --- | --- | --- | --- | --- | --- | --- | --- | --- | --- | --- | --- | --- | --- | --- | --- | --- | --- | --- | --- | --- | --- | --- | --- | --- | --- | --- | --- | --- | --- | --- | --- | --- | --- | --- | --- | --- | --- | --- | --- | --- | --- | --- | --- | --- | --- | --- | --- | --- | --- | --- | --- | --- | --- | --- | --- | --- | --- | --- | --- | --- | --- | --- | --- | --- | --- | --- | --- | --- | --- | --- | --- | --- | --- | --- | --- | --- | --- | --- | --- | --- | --- | --- | --- | --- | --- | --- | --- | --- | --- | --- | --- | --- | --- | --- | --- | --- | --- | --- | --- | --- | --- | --- | --- | --- | --- | --- | --- | --- | --- | --- | --- | --- | --- | --- | --- | --- | --- | --- | --- | --- | --- | --- | --- | --- | --- | --- | --- | --- | --- | --- | --- | --- | --- | --- | --- | --- | --- | --- | --- | --- | --- | --- | --- | --- | --- | --- | --- | --- | --- | --- | --- | --- | --- | --- | --- | --- | --- | --- | --- | --- | --- | --- | --- | --- | --- | --- | --- | --- | --- | --- | --- | --- | --- | --- | --- | --- | --- | --- | --- | --- | --- | --- | --- | --- | --- | --- | --- | --- | --- | --- | --- | --- | --- | --- | --- | --- | --- | --- | --- | --- | --- | --- | --- | --- | --- | --- | --- | --- | --- | --- | --- | --- | --- | --- | --- | --- | --- | --- | --- | --- | --- | --- | --- | --- | --- | --- | --- | --- | --- | --- | --- | --- | --- | --- | --- | --- | --- | --- | --- | --- | --- | --- | --- | --- | --- | --- | --- | --- | --- | --- | --- | --- | --- | --- | --- | --- | --- | --- | --- | --- | --- | --- | --- | --- | --- | --- | --- | --- | --- | --- | --- | --- | --- | --- | --- | --- | --- | --- | --- | --- | --- | --- | --- | --- | --- | --- | --- | --- | --- | --- | --- | --- | --- | --- | --- | --- | --- | --- | --- | --- | --- | --- | --- | --- | --- | --- | --- | --- | --- | --- | --- | --- | --- | --- | --- | --- | --- | --- | --- | --- | --- | --- | --- | --- | --- | --- | --- | --- | --- | --- | --- | --- | --- | --- | --- | --- | --- | --- | --- | --- | --- | --- | --- | --- | --- | --- | --- | --- | --- | --- | --- | --- | --- | --- | --- | --- | --- | --- | --- | --- | --- | --- | --- | --- | --- | --- | --- | --- | --- | --- | --- | --- | --- | --- | --- | --- | --- | --- | --- | --- | --- | --- | --- | --- | --- | --- | --- | --- | --- | --- | --- | --- | --- | --- | --- | --- | --- | --- | --- | --- | --- | --- | --- | --- | --- | --- | --- | --- | --- | --- | --- | --- | --- | --- | --- | --- | --- | --- | --- | --- | --- | --- | --- | --- | --- | --- | --- | --- | --- | --- | --- | --- | --- | --- | --- | --- | --- | --- | --- | --- | --- | --- | --- | --- | --- | --- | --- | --- | --- | --- | --- | --- | --- | --- | --- | --- | --- | --- | --- | --- | --- | --- | --- | --- | --- | --- | --- | --- | --- | --- | --- | --- | --- | --- | --- | --- | --- | --- | --- | --- | --- | --- | --- | --- | --- | --- | --- | --- | --- | --- | --- | --- | --- | --- | --- | --- | --- | --- | --- | --- | --- | --- | --- | --- | --- | --- | --- | --- | --- | --- | --- | --- | --- | --- | --- | --- | --- | --- | --- | --- | --- | --- | --- | --- | --- | --- | --- | --- | --- | --- | --- | --- | --- | --- | --- | --- | --- | --- | --- | --- | --- | --- | --- | --- | --- | --- | --- | --- | --- | --- | --- | --- | --- | --- | --- | --- | --- | --- | --- | --- | --- | --- | --- | --- | --- | --- | --- | --- | --- | --- | --- | --- | --- | --- | --- | --- | --- | --- | --- | --- | --- | --- | --- | --- | --- | --- | --- | --- | --- | --- | --- | --- | --- | --- | --- | --- | --- | --- | --- | --- | --- | --- | --- | --- | --- | --- | --- | --- | --- | --- | --- | --- | --- | --- | --- | --- | --- | --- | --- | --- | --- | --- | --- | --- | --- | --- | --- | --- | --- | --- | --- | --- | --- | --- | --- | --- | --- | --- | --- | --- | --- | --- | --- | --- | --- | --- | --- | --- | --- | --- | --- | --- | --- | --- | --- | --- | --- | --- | --- | --- | --- | --- | --- | --- | --- | --- | --- | --- | --- | --- | --- | --- | --- | --- | --- | --- | --- | --- | --- | --- | --- | --- | --- | --- | --- | --- | --- | --- | --- | --- | --- | --- | --- | --- | --- | --- | --- | --- | --- | --- | --- | --- | --- | --- | --- | --- | --- | --- | --- | --- | --- | --- | --- | --- | --- | --- | --- | --- | --- | --- | --- | --- | --- | --- | --- | --- | --- | --- | --- | --- | --- | --- | --- | --- | --- | --- | --- | --- | --- | --- | --- | --- | --- | --- | --- | --- | --- | --- | --- | --- | --- | --- | --- | --- | --- | --- | --- | --- | --- | --- | --- | --- | --- | --- | --- | --- | --- | --- | --- | --- | --- | --- | --- | --- | --- | --- | --- | --- | --- | --- | --- | --- | --- | --- | --- | --- | --- | --- | --- | --- | --- | --- | --- | --- | --- | --- | --- | --- | --- | --- | --- | --- | --- | --- | --- | --- | --- | --- | --- | --- | --- | --- | --- | --- | --- | --- | --- | --- | --- | --- | --- | --- | --- | --- | --- | --- | --- | --- | --- | --- | --- | --- | --- | --- | --- | --- | --- | --- | --- | --- | --- | --- | --- | --- | --- | --- | --- | --- | --- | --- | --- | --- | --- | --- | --- | --- | --- | --- | --- | --- | --- | --- | --- | --- | --- | --- | --- | --- | --- | --- | --- | --- | --- | --- | --- | --- | --- | --- | --- | --- | --- | --- | --- | --- | --- | --- | --- | --- | --- | --- | --- | --- | --- | --- | --- | --- | --- | --- | --- | --- | --- | --- | --- | --- | --- | --- | --- | --- | --- | --- | --- | --- | --- | --- | --- | --- | --- | --- | --- | --- | --- | --- | --- | --- | --- | --- | --- | --- | --- | --- | --- | --- | --- | --- | --- | --- | --- | --- | --- | --- | --- | --- | --- | --- | --- | --- | --- | --- | --- | --- | --- | --- | --- | --- | --- | --- | --- | --- | --- | --- | --- | --- | --- | --- | --- | --- | --- | --- | --- | --- | --- | --- | --- | --- | --- | --- | --- | --- | --- | --- | --- | --- | --- | --- | --- | --- | --- | --- | --- | --- | --- | --- | --- | --- | --- | --- | --- | --- | --- | --- | --- | --- | --- | --- | --- | --- | --- | --- | --- | --- | --- | --- | --- | --- | --- | --- | --- | --- | --- | --- | --- | --- | --- | --- | --- | --- | --- | --- | --- | --- | --- | --- | --- | --- | --- | --- | --- | --- | --- | --- | --- | --- | --- | --- | --- | --- | --- | --- | --- | --- | --- | --- | --- | --- | --- | --- | --- | --- | --- | --- | --- | --- | --- | --- | --- | --- | --- | --- | --- | --- | --- | --- | --- | --- | --- | --- | --- | --- | --- | --- | --- | --- | --- | --- | --- | --- | --- | --- | --- | --- | --- | --- | --- | --- | --- | --- | --- | --- | --- | --- | --- | --- | --- | --- | --- | --- | --- | --- | --- | --- | --- | --- | --- | --- | --- | --- | --- | --- | --- | --- | --- | --- | --- | --- | --- | --- | --- | --- | --- | --- | --- | --- | --- | --- | --- | --- | --- | --- | --- | --- | --- | --- | --- | --- | --- | --- | --- | --- | --- | --- | --- | --- | --- | --- | --- | --- | --- | --- | --- | --- | --- | --- | --- | --- | --- | --- | --- | --- | --- | --- | --- | --- | --- | --- | --- | --- | --- | --- | --- | --- | --- | --- | --- | --- | --- | --- | --- | --- | --- | --- | --- | --- | --- | --- | --- | --- | --- | --- | --- | --- | --- | --- | --- | --- | --- | --- | --- | --- | --- | --- | --- | --- | --- | --- | --- | --- | --- | --- | --- | --- | --- | --- | --- | --- | --- | --- | --- | --- | --- | --- | --- | --- | --- | --- | --- | --- | --- | --- | --- | --- | --- | --- | --- | --- | --- | --- | --- | --- | --- | --- | --- | --- | --- | --- | --- | --- | --- | --- | --- | --- | --- | --- | --- | --- | --- | --- | --- | --- | --- | --- | --- | --- | --- | --- | --- | --- | --- | --- | --- | --- | --- | --- | --- | --- | --- | --- | --- | --- | --- | --- | --- | --- | --- | --- | --- | --- | --- | --- | --- | --- | --- | --- | --- | --- | --- | --- | --- | --- | --- | --- | --- | --- | --- | --- | --- | --- | --- | --- | --- | --- | --- | --- | --- | --- | --- | --- | --- | --- | --- | --- | --- | --- | --- | --- | --- | --- | --- | --- | --- | --- | --- | --- | --- | --- | --- | --- | --- | --- | --- | --- | --- | --- | --- | --- | --- | --- | --- | --- | --- | --- | --- | --- | --- | --- | --- | --- | --- | --- | --- | --- | --- | --- | --- | --- | --- | --- | --- | --- | --- | --- | --- | --- | --- | --- | --- | --- | --- | --- | --- | --- | --- | --- | --- | --- | --- | --- | --- | --- | --- | --- | --- | --- | --- | --- | --- | --- | --- | --- | --- | --- | --- | --- | --- | --- | --- | --- | --- | --- | --- | --- | --- | --- | --- | --- | --- | --- | --- | --- | --- | --- | --- | --- | --- | --- | --- | --- | --- | --- | --- | --- | --- | --- | --- | --- | --- | --- | --- | --- | --- | --- | --- | --- | --- | --- | --- | --- | --- | --- | --- | --- | --- | --- | --- | --- | --- | --- | --- | --- | --- | --- | --- | --- | --- | --- | --- | --- | --- | --- | --- | --- | --- | --- | --- | --- | --- | --- | --- | --- | --- | --- | --- | --- | --- | --- | --- | --- | --- | --- | --- | --- | --- | --- | --- | --- | --- | --- | --- | --- | --- | --- | --- | --- | --- | --- | --- | --- | --- | --- | --- | --- | --- | --- | --- | --- | --- | --- | --- | --- | --- | --- | --- | --- | --- | --- | --- | --- | --- | --- | --- | --- | --- | --- | --- | --- | --- | --- | --- | --- | --- | --- | --- | --- | --- | --- | --- | --- | --- | --- | --- | --- | --- | --- | --- | --- | --- | --- | --- | --- | --- | --- | --- | --- | --- | --- | --- | --- | --- | --- | --- | --- | --- | --- | --- | --- | --- | --- | --- | --- | --- | --- | --- | --- | --- | --- | --- | --- | --- | --- | --- | --- | --- | --- | --- | --- | --- | --- | --- | --- | --- | --- | --- | --- | --- | --- | --- | --- | --- | --- | --- | --- | --- | --- | --- | --- | --- | --- | --- | --- | --- | --- | --- | --- | --- | --- | --- | --- | --- | --- | --- | --- | --- | --- | --- | --- | --- | --- | --- | --- | --- | --- | --- | --- | --- | --- | --- | --- | --- | --- | --- | --- | --- | --- | --- | --- | --- | --- | --- | --- | --- | --- | --- | --- | --- | --- | --- | --- | --- | --- | --- | --- | --- | --- | --- | --- | --- | --- | --- | --- | --- | --- | --- | --- | --- | --- | --- | --- | --- | --- | --- | --- | --- | --- | --- | --- | --- | --- | --- | --- | --- | --- | --- | --- | --- | --- | --- | --- | --- | --- | --- | --- | --- | --- | --- | --- | --- | --- | --- | --- | --- | --- | --- | --- | --- | --- | --- | --- | --- | --- | --- | --- | --- | --- | --- | --- | --- | --- | --- | --- | --- | --- | --- | --- | --- | --- | --- | --- | --- | --- | --- | --- | --- | --- | --- | --- | --- | --- | --- | --- | --- | --- | --- | --- | --- | --- | --- | --- | --- | --- | --- | --- | --- | --- | --- | --- | --- | --- | --- | --- | --- | --- | --- | --- | --- | --- | --- | --- | --- | --- | --- | --- | --- | --- | --- | --- | --- | --- | --- | --- | --- | --- | --- | --- | --- | --- | --- | --- | --- | --- | --- | --- | --- | --- | --- | --- | --- | --- | --- | --- | --- | --- | --- | --- | --- | --- | --- | --- | --- | --- | --- | --- | --- | --- | --- | --- | --- | --- | --- | --- | --- | --- | --- | --- | --- | --- | --- | --- | --- | --- | --- | --- | --- | --- | --- | --- | --- | --- | --- | --- | --- | --- | --- | --- | --- | --- | --- | --- | --- | --- | --- | --- | --- | --- | --- | --- | --- | --- | --- | --- | --- | --- | --- | --- | --- | --- | --- | --- | --- | --- | --- | --- | --- | --- | --- | --- | --- | --- | --- | --- | --- | --- | --- | --- | --- | --- | --- | --- | --- | --- | --- | --- | --- | --- | --- | --- | --- | --- | --- | --- | --- | --- | --- | --- | --- | --- | --- | --- | --- | --- | --- | --- | --- | --- | --- | --- | --- | --- | --- | --- | --- | --- | --- | --- | --- | --- | --- | --- | --- | --- | --- | --- | --- | --- | --- | --- | --- | --- | --- | --- | --- | --- | --- | --- | --- | --- | --- | --- | --- | --- | --- | --- | --- | --- | --- | --- | --- | --- | --- | --- | --- | --- | --- | --- | --- | --- | --- | --- | --- | --- | --- | --- | --- | --- | --- | --- | --- | --- | --- | --- | --- | --- | --- | --- | --- | --- | --- | --- | --- | --- | --- | --- | --- | --- | --- | --- | --- | --- | --- | --- | --- | --- | --- | --- | --- | --- | --- | --- | --- | --- | --- | --- | --- | --- | --- | --- | --- | --- | --- | --- | --- | --- | --- | --- | --- | --- | --- | --- | --- | --- | --- | --- | --- | --- | --- | --- | --- | --- | --- | --- | --- | --- | --- | --- | --- | --- | --- | --- | --- | --- | --- | --- | --- | --- | --- | --- | --- | --- | --- | --- | --- | --- | --- | --- | --- | --- | --- | --- | --- | --- | --- | --- | --- | --- | --- | --- | --- | --- | --- | --- | --- | --- | --- | --- | --- | --- | --- | --- | --- | --- | --- | --- | --- | --- | --- | --- | --- | --- | --- | --- | --- | --- | --- | --- | --- | --- | --- | --- | --- | --- | --- | --- | --- | --- | --- | --- | --- | --- | --- | --- | --- | --- | --- | --- | --- | --- | --- | --- | --- | --- | --- | --- | --- | --- | --- | --- | --- | --- | --- | --- | --- | --- | --- | --- | --- | --- | --- | --- | --- | --- | --- | --- | --- | --- | --- | --- | --- | --- | --- | --- | --- | --- | --- | --- | --- | --- | --- | --- | --- | --- | --- | --- | --- | --- | --- | --- | --- | --- | --- | --- | --- | --- | --- | --- | --- | --- | --- | --- | --- | --- | --- | --- | --- | --- | --- | --- | --- | --- | --- | --- | --- | --- | --- | --- | --- | --- | --- | --- | --- | --- | --- | --- | --- | --- | --- | --- | --- | --- | --- | --- | --- | --- | --- | --- | --- | --- | --- | --- | --- | --- | --- | --- | --- | --- | --- | --- | --- | --- | --- | --- | --- | --- | --- | --- | --- | --- | --- | --- | --- | --- | --- | --- | --- | --- | --- | --- | --- | --- | --- | --- | --- | --- | --- | --- | --- | --- | --- | --- | --- | --- | --- | --- | --- | --- | --- | --- | --- | --- | --- | --- | --- | --- | --- | --- | --- | --- | --- | --- | --- | --- | --- | --- | --- | --- | --- | --- | --- | --- | --- | --- | --- | --- | --- | --- | --- | --- | --- | --- | --- | --- | --- | --- | --- | --- | --- | --- | --- | --- | --- | --- | --- | --- | --- | --- | --- | --- | --- | --- | --- | --- | --- | --- | --- | --- | --- | --- | --- | --- | --- | --- | --- | --- | --- | --- | --- | --- | --- | --- | --- | --- | --- | --- | --- | --- | --- | --- | --- | --- | --- | --- | --- | --- | --- | --- | --- | --- | --- | --- | --- | --- | --- | --- | --- | --- | --- | --- | --- | --- | --- | --- | --- | --- | --- | --- | --- | --- | --- | --- | --- | --- | --- | --- | --- | --- | --- | --- | --- | --- | --- | --- | --- | --- | --- | --- | --- | --- | --- | --- | --- | --- | --- | --- | --- | --- | --- | --- | --- | --- | --- | --- | --- | --- | --- | --- | --- | --- | --- | --- | --- | --- | --- | --- | --- | --- | --- | --- | --- | --- | --- | --- | --- | --- | --- | --- | --- | --- | --- | --- | --- | --- | --- | --- | --- | --- | --- | --- | --- | --- | --- | --- | --- | --- | --- | --- | --- | --- | --- | --- | --- | --- | --- | --- | --- | --- | --- | --- | --- | --- | --- | --- | --- | --- | --- | --- | --- | --- | --- | --- | --- | --- | --- | --- | --- | --- | --- | --- | --- | --- | --- | --- | --- | --- | --- | --- | --- | --- | --- | --- | --- | --- | --- | --- | --- | --- | --- | --- | --- | --- | --- | --- | --- | --- | --- | --- | --- | --- | --- | --- | --- | --- | --- | --- | --- | --- | --- | --- | --- | --- | --- | --- | --- | --- | --- | --- | --- | --- | --- | --- | --- | --- | --- | --- | --- | --- | --- | --- | --- | --- | --- | --- | --- | --- | --- | --- | --- | --- | --- | --- | --- | --- | --- | --- | --- | --- | --- | --- | --- | --- | --- | --- | --- | --- | --- | --- | --- | --- | --- | --- | --- | --- | --- | --- | --- | --- | --- | --- | --- | --- | --- | --- | --- | --- | --- | --- | --- | --- | --- | --- | --- | --- | --- | --- | --- | --- | --- | --- | --- | --- | --- | --- | --- | --- | --- | --- | --- | --- | --- | --- | --- | --- | --- | --- | --- | --- | --- | --- | --- | --- | --- | --- | --- | --- | --- | --- | --- | --- | --- | --- | --- | --- | --- | --- | --- | --- | --- | --- | --- | --- | --- | --- | --- | --- | --- | --- | --- | --- | --- | --- | --- | --- | --- | --- | --- | --- | --- | --- | --- | --- | --- | --- | --- | --- | --- | --- | --- | --- | --- | --- | --- | --- | --- | --- | --- | --- | --- | --- | --- | --- | --- | --- | --- | --- | --- | --- | --- | --- | --- | --- | --- | --- | --- | --- | --- | --- | --- | --- | --- | --- | --- | --- | --- | --- | --- | --- | --- | --- | --- | --- | --- | --- | --- | --- | --- | --- | --- | --- | --- | --- | --- | --- | --- | --- | --- | --- | --- | --- | --- | --- | --- | --- | --- | --- | --- | --- | --- | --- | --- | --- | --- | --- | --- | --- | --- | --- | --- | --- | --- | --- | --- | --- | --- | --- | --- | --- | --- | --- | --- | --- | --- | --- | --- | --- | --- | --- | --- | --- | --- | --- | --- | --- | --- | --- | --- | --- | --- | --- | --- | --- | --- | --- | --- | --- | --- | --- | --- | --- | --- | --- | --- | --- | --- | --- | --- | --- | --- | --- | --- | --- | --- | --- | --- | --- | --- | --- | --- | --- | --- | --- | --- | --- | --- | --- | --- | --- | --- | --- | --- | --- | --- | --- | --- | --- | --- | --- | --- | --- | --- | --- | --- | --- | --- | --- | --- | --- | --- | --- | --- | --- | --- | --- | --- | --- | --- | --- | --- | --- | --- | --- | --- | --- | --- | --- | --- | --- | --- | --- | --- | --- | --- | --- | --- | --- | --- | --- | --- | --- | --- | --- | --- | --- | --- | --- | --- | --- | --- | --- | --- | --- | --- | --- | --- | --- | --- | --- | --- | --- | --- | --- | --- | --- | --- | --- | --- | --- | --- | --- | --- | --- | --- | --- | --- | --- | --- | --- | --- | --- | --- | --- | --- | --- | --- | --- | --- | --- | --- | --- | --- | --- | --- | --- | --- | --- | --- | --- | --- | --- | --- | --- | --- | --- | --- | --- | --- | --- | --- | --- | --- | --- | --- | --- | --- | --- | --- | --- | --- | --- | --- | --- | --- | --- | --- | --- | --- | --- | --- | --- | --- | --- | --- | --- | --- | --- | --- | --- | --- | --- | --- | --- | --- | --- | --- | --- | --- | --- | --- | --- | --- | --- | --- | --- | --- | --- | --- | --- | --- | --- | --- | --- | --- | --- | --- | --- | --- | --- | --- | --- | --- | --- | --- | --- | --- | --- | --- | --- | --- | --- | --- | --- | --- | --- | --- | --- | --- | --- | --- | --- | --- | --- | --- | --- | --- | --- | --- | --- | --- | --- | --- | --- | --- | --- | --- | --- | --- | --- | --- | --- | --- | --- | --- | --- | --- | --- | --- | --- | --- | --- | --- | --- | --- | --- | --- | --- | --- | --- | --- | --- | --- | --- | --- | --- | --- | --- | --- | --- | --- | --- | --- | --- | --- | --- | --- | --- | --- | --- | --- | --- | --- | --- | --- | --- | --- | --- | --- | --- | --- | --- | --- | --- | --- | --- | --- | --- | --- | --- | --- | --- | --- | --- | --- | --- | --- | --- | --- | --- | --- | --- | --- | --- | --- | --- | --- | --- | --- | --- | --- | --- | --- | --- | --- | --- | --- | --- | --- | --- | --- | --- | --- | --- | --- | --- | --- | --- | --- | --- | --- | --- | --- | --- | --- | --- | --- | --- | --- | --- | --- | --- | --- | --- | --- | --- | --- | --- | --- | --- | --- | --- | --- | --- | --- | --- | --- | --- | --- | --- | --- | --- | --- | --- | --- | --- | --- | --- | --- | --- | --- | --- | --- | --- | --- | --- | --- | --- | --- | --- | --- | --- | --- | --- | --- | --- | --- | --- | --- | --- | --- | --- | --- | --- | --- | --- | --- | --- | --- | --- | --- | --- | --- | --- | --- | --- | --- | --- | --- | --- | --- | --- | --- | --- | --- | --- | --- | --- | --- | --- | --- | --- | --- | --- | --- | --- | --- | --- | --- | --- | --- | --- | --- | --- | --- | --- | --- | --- | --- | --- | --- | --- | --- | --- | --- | --- | --- | --- | --- | --- | --- | --- | --- | --- | --- | --- | --- | --- | --- | --- | --- | --- | --- | --- | --- | --- | --- | --- | --- | --- | --- | --- | --- | --- | --- | --- | --- | --- | --- | --- | --- | --- | --- | --- | --- | --- | --- | --- | --- | --- | --- | --- | --- | --- | --- | --- | --- | --- | --- | --- | --- | --- | --- | --- | --- | --- | --- | --- | --- | --- | --- | --- | --- | --- | --- | --- | --- | --- | --- | --- | --- | --- | --- | --- | --- | --- | --- | --- | --- | --- | --- | --- | --- | --- | --- | --- | --- | --- | --- | --- | --- | --- | --- | --- | --- | --- | --- | --- | --- | --- | --- | --- | --- | --- | --- | --- | --- | --- | --- | --- | --- | --- | --- | --- | --- | --- | --- | --- | --- | --- | --- | --- | --- | --- | --- | --- | --- | --- | --- | --- | --- | --- | --- | --- | --- | --- | --- | --- | --- | --- | --- | --- | --- | --- | --- | --- | --- | --- | --- | --- | --- | --- | --- | --- | --- | --- | --- | --- | --- | --- | --- | --- | --- | --- | --- | --- | --- | --- | --- | --- | --- | --- | --- | --- | --- | --- | --- | --- | --- | --- | --- | --- | --- | --- | --- | --- | --- | --- | --- | --- | --- | --- | --- | --- | --- | --- | --- | --- | --- | --- | --- | --- | --- | --- | --- | --- | --- | --- | --- | --- | --- | --- | --- | --- | --- | --- | --- | --- | --- | --- | --- | --- | --- | --- | --- | --- | --- | --- | --- | --- | --- | --- | --- | --- | --- | --- | --- | --- | --- | --- | --- | --- | --- | --- | --- | --- | --- | --- | --- | --- | --- | --- | --- | --- | --- | --- | --- | --- | --- | --- | --- | --- | --- | --- | --- | --- | --- | --- | --- | --- | --- | --- | --- | --- | --- | --- | --- | --- | --- | --- | --- | --- | --- | --- | --- | --- | --- | --- | --- | --- | --- | --- | --- | --- | --- | --- | --- | --- | --- | --- | --- | --- | --- | --- | --- | --- | --- | --- | --- | --- | --- | --- | --- | --- | --- | --- | --- | --- | --- | --- | --- | --- | --- | --- | --- | --- | --- | --- | --- | --- | --- | --- | --- | --- | --- | --- | --- | --- | --- | --- | --- | --- | --- | --- | --- | --- | --- | --- | --- | --- | --- | --- | --- | --- | --- | --- | --- | --- | --- | --- | --- | --- | --- | --- | --- | --- | --- | --- | --- | --- | --- | --- | --- | --- | --- | --- | --- | --- | --- | --- | --- | --- | --- | --- | --- | --- | --- | --- | --- | --- | --- | --- | --- | --- | --- | --- | --- | --- | --- | --- | --- | --- | --- | --- | --- | --- | --- | --- | --- | --- | --- | --- | --- | --- | --- | --- | --- | --- | --- | --- | --- | --- | --- | --- | --- | --- | --- | --- | --- | --- | --- | --- | --- | --- | --- | --- | --- | --- | --- | --- | --- | --- | --- | --- | --- | --- | --- | --- | --- | --- | --- | --- | --- | --- | --- | --- | --- | --- | --- | --- | --- | --- | --- | --- | --- | --- | --- | --- | --- | --- | --- | --- | --- | --- | --- | --- | --- | --- | --- | --- | --- | --- | --- | --- | --- | --- | --- | --- | --- | --- | --- | --- | --- | --- | --- | --- | --- | --- | --- | --- | --- | --- | --- | --- | --- | --- | --- | --- | --- | --- | --- | --- | --- | --- | --- | --- | --- | --- | --- | --- | --- | --- | --- | --- | --- | --- | --- | --- | --- | --- | --- | --- | --- | --- | --- | --- | --- | --- | --- | --- | --- | --- | --- | --- | --- | --- | --- | --- | --- | --- | --- | --- | --- | --- | --- | --- | --- | --- | --- | --- | --- | --- | --- | --- | --- | --- | --- | --- | --- | --- | --- | --- | --- | --- | --- | --- | --- | --- | --- | --- | --- | --- | --- | --- | --- | --- | --- | --- | --- | --- | --- | --- | --- | --- | --- | --- | --- | --- | --- | --- | --- | --- | --- | --- | --- | --- | --- | --- | --- | --- | --- | --- | --- | --- | --- | --- | --- | --- | --- | --- | --- | --- | --- | --- | --- | --- | --- | --- | --- | --- | --- | --- | --- | --- | --- | --- | --- | --- | --- | --- | --- | --- | --- | --- | --- | --- | --- | --- | --- | --- | --- | --- | --- | --- | --- | --- | --- | --- | --- | --- | --- | --- | --- | --- | --- | --- | --- | --- | --- | --- | --- | --- | --- | --- | --- | --- | --- | --- | --- | --- | --- | --- | --- | --- | --- | --- | --- | --- | --- | --- | --- | --- | --- | --- | --- | --- | --- | --- | --- | --- | --- | --- | --- | --- | --- | --- | --- |
